# Few-shot deployment of pretrained MRI transformers in brain imaging tasks

**DOI:** 10.3389/frai.2026.1771088

**Published:** 2026-04-01

**Authors:** Mengyu Li, Guoyao Shen, Chad W. Farris, Xin Zhang

**Affiliations:** 1 Department of Mechanical Engineering, Boston University, Boston, MA, United States; 2 The Photonics Center, Boston University, Boston, MA, United States; 3 Department of Radiology, Boston University Chobanian & Avedisian School of Medicine, Boston, MA, United States; 4 Department of Radiology, Boston Medical Center, Boston, MA, United States; 5 Department of Electrical and Computer Engineering, Boston University, Boston, MA, United States; 6 Department of Biomedical Engineering, Boston University, Boston, MA, United States; 7 Division of Materials Science and Engineering, Boston University, Boston, MA, United States; 8 Rafik B. Hariri Institute for Computing and Computational Science & Engineering, Boston University, Boston, MA, United States

**Keywords:** brain MRI, classification, deep learning, few-shot learning, medical imaging, pretraining and fine-tuning, segmentation, transformer

## Abstract

**Introduction:**

Transformer-based deep learning has shown great potential in medical imaging, but its real-world applicability remains limited due to the scarcity of annotated data. This study aims to develop a practical framework for the few-shot deployment of pretrained MRI transformers across diverse brain imaging tasks.

**Methods:**

We employ a Masked Autoencoder (MAE) pretraining strategy on a large-scale, multi-cohort brain MRI dataset comprising over 31 million 2D slices to learn transferable representations. For classification tasks, a frozen MAE encoder with a lightweight linear head (MAE-classify) is used. For segmentation, we propose MAE-FUnet, a hybrid architecture that fuses pretrained MAE embeddings with multi-scale CNN features. Extensive evaluations are conducted on multiple datasets, including NACC, ADNI, OASIS, NFBS, SynthStrip, and MRBrainS18, under controlled few-shot settings.

**Results:**

The proposed framework achieves state-of-the-art performance in MRI sequence classification, reaching an accuracy of 99.24% with only 6,152 trainable parameters. For segmentation tasks, MAE-FUnet consistently outperforms strong baselines, achieving superior Dice and IoU scores across skull stripping and multi-class anatomical segmentation benchmarks. The model also demonstrates enhanced robustness and stability under data-limited conditions, with lower performance variance compared to competing methods.

**Discussion:**

These results highlight the effectiveness of pretrained MAE representations for few-shot medical imaging tasks. The proposed framework enables efficient, scalable, and adaptable deployment of transformer-based models in data-constrained clinical environments. The fusion of global transformer embeddings with local CNN features provides a generalizable design paradigm for a wide range of medical imaging applications.

## 1 Introduction

Recent advances in deep learning have made remarkable progress in medical imaging interpretation (Shin et al., 2016; Litjens et al., 2017; Cai et al., 2020; Singh et al., 2020; Suganyadevi et al., 2022). A wide range of deep learning architectures has been explored for medical analysis, including CNNs (Ronneberger et al., 2015; Rajpurkar et al., 2018), MLPs (Yan et al., 2022), transformers (Lin and Heckel, 2022; Shen et al., 2024a), and diffusion models (Xie and Li, 2022; Müller-Franzes et al., 2023; Shen et al., 2024b). Transformer-based architectures, originally developed in the NLP community (Vaswani et al., 2017; Devlin et al., 2019; Brown et al., 2020; Patwardhan et al., 2023), have demonstrated strong generalization capabilities across vision and multimodal tasks, motivating their adoption in a wide range of medical imaging applications (Henry et al., 2022; Azad et al., 2024). In medical imaging, transformers have shown particular effectiveness in tasks such as brain structure segmentation, lesion detection, and image classification (Hatamizadeh et al., 2022; Flosdorf et al., 2024; Gao et al., 2024). Their long-range attention mechanisms enable modeling of global spatial context much more effectively than conventional CNNs or MLPs. However, the large number of trainable parameters and attention-based structure of transformers often require substantial amounts of training data. As a result, deploying transformer-based models in real-world clinical settings remains challenging when annotated datasets are limited.

In clinical neuroimaging, data scarcity remains a common obstacle due to privacy restrictions, annotation cost, and heterogeneity across imaging protocols. Existing approaches that aim to mitigate data scarcity in clinical neuroimaging typically fall into several categories: meta-learning approaches that involve complex fine-tuning procedures (Gama et al., 2022; Lin et al., 2023; Oliveira et al., 2024; Tang F. et al., 2025; Tang S. et al., 2025), methods restricted to specific downstream tasks (Ouyang et al., 2020; Kim et al., 2022), techniques limited to well-established 3D MRI datasets (Chen et al., 2019), or frameworks that rely on manual input (Xie et al., 2024). These limitations can be addressed by leveraging vision transformers pretrained on large-scale, universal 2D datasets, which can be deployed to various downstream tasks under few-shot conditions. One of the most widely adopted pretraining methods for vision transformers is the Masked Autoencoder (MAE) (He et al., 2022), which initiates pretraining by reconstructing the masked patches in 2D images. In our work, we focus on utilizing an MAE-style pretraining strategy on a large 2D brain MRI cohort to obtain a pretrained vision transformer architecture as a strong and transferable initialization for downstream tasks. Unlike models trained specifically for a single task or dataset, pretrained vision transformers can be repurposed with appropriate fine-tuning designs, offering promising performance even in small-sample regimes. We systematically evaluate the MAE-based, few-shot architectures on non-pathological brain MRI tasks, including both classification and segmentation across diverse datasets. In our experiment, the few-shot setting is explicitly defined by controlled experiments under varying degrees of data scarcity.

Our approach targets multiple key benchmarks spanning both classification and segmentation tasks in brain MRI. Because different downstream tasks impose distinct modeling requirements, we construct task-specific model variants to explore effective strategies for reusing the pretrained MAE embeddings. For classification, we investigate the performance of a lightweight MAE-based classifier in discriminating MRI sequence modalities such as T1, T2, Fluid-Attenuated Inversion Recovery (FLAIR), Proton Density (PD), diffusion imaging (Diffusion Tensor Imaging (DTI) and Diffusion Weighted Imaging (DWI)). For segmentation, we assess an MAE variant that fuses the transformer’s latent-space embeddings with the U-Net features across multiple layers. This architecture, referred to as MAE-Fused U-Net (MAE-FUnet), is rigorously tested on both skull stripping and multi-class anatomical segmentation across multiple datasets, including Neurofeedback Skull-stripped (NFBS) (Puccio et al., 2016), SynthStrip (Hoopes et al., 2022), and MRBrainS18 (Kuijf et al., 2024). For each task domain, the MAE-based variants are compared against well-established baselines. For classification, baselines include EfficientNetV2 (Tan and Le, 2021), ResNet (He et al., 2016), and MedViT (Manzari et al., 2023). For segmentation, we compare against Swin-Unet (Cao et al., 2022), U-Net, TransUNet (Chen J. et al., 2021), and SegFormer (Xie et al., 2021). Across these benchmarks, the pretrained MAE-based models demonstrate strong and often superior performance under few-shot scenarios.

This study highlights the practicality and scalability of deploying pretrained transformers in real-world, data-limited brain imaging pipelines. In contrast to prior studies that focus on efficient fine-tuning (Dhinagar et al., 2025), non–few-shot deployment settings (Zhou et al., 2023; Tang F. et al., 2025; Tang S. et al., 2025), or pretraining within narrow modality or task scopes (Wald et al., 2025; Zhuang et al., 2025), practical clinical deployment imposes additional requirements. Specifically, it requires (i) pretraining that is robust to heterogeneous MRI protocols and sequence types, and (ii) task-aware adaptation strategies that differ substantially between high-level analytical tasks and low-level dense prediction tasks (Shen et al., 2023; Chen H. et al., 2021) under extreme data scarcity. To address these deployment-centric gaps, we combine large-scale, multi-cohort, multi-sequence MRI MAE pretraining with systematic, task-aware downstream designs, instantiating the same MAE backbone as *MAE-classify* for lightweight classification and *MAE-FUnet* for fusion-based segmentation. Together with controlled few-shot evaluation protocols spanning various task domains, this framework demonstrates how a single pretrained transformer can be reliably repurposed into efficient, customizable models suitable for clinical environments where annotated data are limited and target tasks are narrowly defined.

## 2 Materials and methods

### 2.1 Dataset details

#### 2.1.1 Pretraining datasets

**Figure 1:**
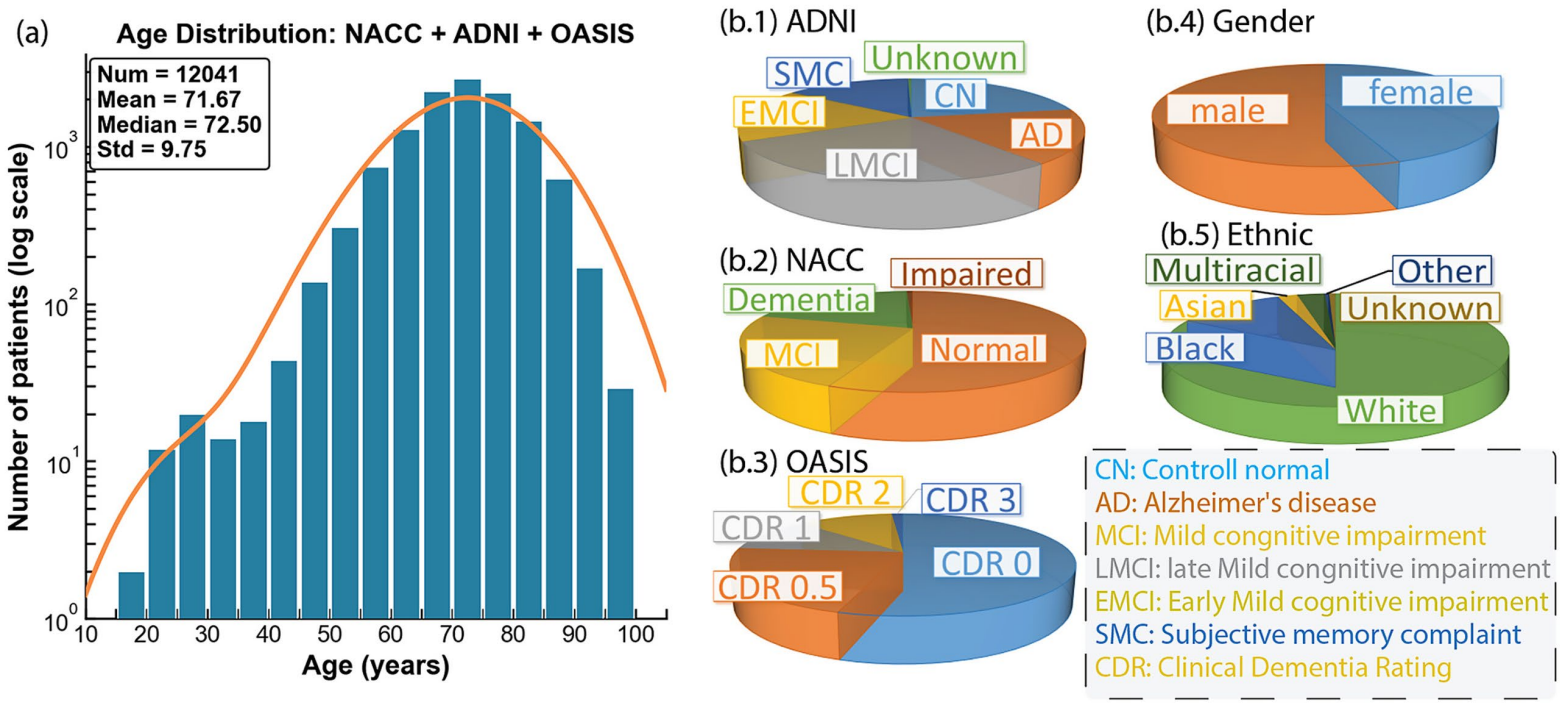
**(a)** Combined pretrain dataset’s age distribution from NACC, ADNI, and OASIS. **(b)** Distribution of ADNI, NACC, OASIS baseline diagnosis, total gender, and ethnic groups.

To invoke larger-scale pretraining, we first build a comprehensive dataset consisting of five brain MRI cohorts with three brain pathology-related datasets: National Alzheimer’s Coordinating Center (NACC), Open Access Series of Imaging Studies (OASIS), Alzheimer’s Disease Neuroimaging Initiative (ADNI); and two non-pathological datasets: FastMRI (Zbontar et al., 2018) and RadImageNet (Mei et al., 2022). Each consists of a large amount of brain MRI with sequences spanning T1, T2, FLAIR, PD, and DWI. After excluding non-brain MRI regions, such as knee MRI in FastMRI, the final dataset is a large brain MRI dataset with 31 million 2D slices in total. As demonstrated in Table 1, the three brain pathology-related datasets contribute to the majority of the MRI slices. Their demographic distributions, including age, ethnic groups, gender, and diagnosis, are illustrated in Figure 1. The detailed protocols and standards for MRI collection during pretraining are outlined below.

**Table 1:** MRI slices and patient numbers in each pretrain dataset.

Dataset	NACC	ADNI	OASIS	RAD	FastMRI
# Slice	8.5 million	12 million	10 million	44,671	97,900
# Patients	7,640	2,430	1,377	–	–

##### 2.1.1.1 National Alzheimer’s Coordinating Center

The NACC dataset, maintained by the National Alzheimer’s Coordinating Center, is a large, publicly available database used extensively in neuroscience and medical research. The version used in this study is the latest Uniform Data Set (UDS): Version 3 (UDSv3) with the Neuropathology Data Set (NP) currently at Version 11. Overall, it includes more than 54,000 patients and more than 200,000 clinical assessments, with MRI records spanning the years 2005 to 2024. In our study, we include only MRI sessions to construct a dataset comprising sequence modalities: T1, T2, FLAIR, DWI, DTI, and PD. The total number of 2D MRI slices from the NACC dataset is 8.5 million.

##### 2.1.1.2 Alzheimer’s Disease Neuroimaging Initiative

Data used in the preparation of this article were obtained from the Alzheimer’s Disease Neuroimaging Initiative (ADNI) database (adni.loni.usc.edu). The ADNI was launched in 2003 as a public-private partnership, led by Principal Investigator Michael W. Weiner, MD. The primary goal of ADNI has been to test whether serial magnetic resonance imaging (MRI), positron emission tomography (PET), other biological markers, and clinical and neuropsychological assessment can be combined to measure the progression of mild cognitive impairment (MCI) and early Alzheimer’s disease (AD). The dataset used in this study is a combined cohort from all five versions: ADNI1, ADNI2, ADNI3, ADNI4, and ADNIDOD. The dataset primarily focused on structural MRI, including T1, T2, FLAIR, and DWI, with a total of 12 million 2D MRI slices.

##### 2.1.1.3 Open Access Series of Imaging Studies

OASIS is a public neuroimaging dataset designed to support research in aging, Alzheimer’s disease, and neurodegeneration. The original release was developed by the Marcus Institute for Aging Research at Washington University. The dataset includes MRI scans, cognitive assessments, clinical metadata, and other longitudinal data. The MRI scans for our study are filtered from all 4 versions: OASIS-1, OASIS-2, OASIS-3, and OASIS-4 (in progress), which provide a total of 10 million 2D MRI slices across all sequence modalities.

##### 2.1.1.4 RadImageNet

RadImageNet is a large-scale radiology-specific image dataset created to support deep learning research in medical imaging, particularly in radiographic modalities like CT, MRI, and X-ray. Within this study, we pick only brain-related MRIs across categories of white matter changes, pituitary lesions, chronic infarction, normal samples, etc. The entire dataset size of 2D MRI slices is 44,671.

##### 2.1.1.5 FastMRI

FastMRI is a large-scale, open-source dataset and research initiative developed by Facebook AI Research (FAIR) in collaboration with NYU Langone Health, aimed at accelerating MRI reconstruction using deep learning. In our case, only brain-related multi-coil series are selected from sequences T1, T2, and FLAIR. The total number of MRI scans across the train, test, and validation subsets yields 97,900 2D MRI slices.

#### 2.1.2 Fine-tuning dataset

To test the generalization ability of the pretrained model, we conduct few-shot fine-tuning tests not only on subsets of the pretrained datasets, but also on fully independent datasets such as NFBS, SynthStrip, and MRBrainS18 across classification and segmentation tasks. Since each dataset has a different number of sequence types (e.g., T1, T2, FLAIR), we evaluate few-shot learning performance using a controlled sampling strategy in which the training set size is incrementally increased for each available sequence. The overall train/test dataset splits for three fine-tuning tasks: sequence detection, skull stripping, and multi-class anatomical segmentation, are summarized in Table 2. Task-specific few-shot protocols are adopted: slice-level sampling for classification, sparse-slice sampling for skull stripping, and volume-level sampling for anatomical segmentation. To prevent data leakage, all MRI scans included in the few-shot datasets are strictly excluded from the pretraining datasets. The detailed methods for building the fine-tuning datasets are introduced below.

**Table 2:** Train and test split of fine-tuning datasets for tasks including sequence detection, skull stripping, and multi-class anatomical segmentation.

	Train							Test
Sequence detection								
NACC + ADNI + OASIS	Train (Few-shot training size)							Test (Full dataset)
# Slice/sequence/dataset	10	20	30		50	100		10,000–20,000
# Patients/sequence/dataset	1	2	3		5	10		300
Skull stripping								
NFBS	Train (Few-shot training size)							Test (Full dataset)
# Sample strip/sequence	4	5	6	7	8	9	10	–
# Slice/sequence	56	44	37	32	28	24	22	23,408
# Patients/sequence	1	1	1	1	1	1	1	63
SynthStrip	Train (Few-shot training size)							Test (Full dataset)
# Sample Strip/sequence	4	5	6	7	8	9	10	–
# Slice/sequence	56	44	37	32	28	24	22	12,623
# Patients/sequence	1	1	1	1	1	1	1	30 (16 for FLAIR)
Multi-class anatomical segmentation								
MRBrainS18	Train (Few-shot training size)							Test (Full dataset)
# 3D Volume/sequence	7							23
# Slice/sequence	257							864
# Patients/sequence	7							23
NACC	Train (Few-shot training size)							Test (Full dataset)
# 3D Volume/sequence	10	20	30		50	100		2,434
# Slice/sequence	1,816	2,789	4,164		6,359	16,887		408,266
# Patients/sequence	6	13	18		36	63		1,190

The train dataset increments at a pace customized for each task/dataset within its available sequence.

##### 2.1.2.1 Sequence detection dataset

The dataset built for sequence detection is derived from NACC, ADNI, and OASIS. For each cohort, we start by randomly selecting 1 patient’s MRI for each sequence (T1, T2, T2 FLAIR, PD, T2* (T2 Hemorrhage in particular), T2 SWI, and DTI/DWI), then randomly sample 10 slices from the selected MRI. Therefore, the dataset comprises 10 slices per sequence, per cohort. Then the training dataset gradually increases, with random sampling increasing from 1 patient to 10 patients, yielding training sizes ranging from 10 slices/sequence/dataset to 100 slices/sequence/dataset. The test dataset is held fixed and comprises MRI scans from 300 patients per sequence. Due to variability in acquisition protocols and scan coverage across subjects, the total number of slices per sequence in the test dataset ranges from approximately 10,000 to 20,000 per cohort. In total, the test dataset contains approximately 60,000 slices for each of the T1, T2, T2-FLAIR, T2-SWI, and DTI/DWI sequences, and approximately 40,000 slices for each of the PD and T2* sequences. All MRI scans (approximately 390,000 slices) used for both training and testing have been pre-excluded from pretraining datasets to avoid data leakage.

##### 2.1.2.2 Skull stripping dataset

The skull stripping task is tested on two cohorts: NFBS and SynthStrip, both of which include hand-annotated skull stripping masks. The details of the cohorts and the methods used to build the fine-tuning datasets are presented below.

###### 2.1.2.2.1 Neurofeedback skull-stripped repository

The NFBS dataset is a repository of high-quality, manually skull-stripped T1-weighted anatomical MRI scans designed as a gold standard for brain extraction (skull-stripping) in neuroimaging research. As part of the Enhanced Rockland Sample Neurofeedback Study, it contains T1-weighted MRI scans in NIfTI format from 125 participants, aged 21 to 45, with a variety of clinical and subclinical psychiatric symptoms. In our skull stripping experiment, we split it into a training dataset of randomly sampled 1 patient and a testing dataset of 63 patients.

###### 2.1.2.2.2 SynthStrip

The SynthStrip dataset is an open-access collection designed for developing and evaluating skull-stripping (brain extraction) tools across a wide range of imaging modalities, populations, and protocols. It was originally created to test FreeSurfer software with 622 full-head 3D scans consisting of MRI, CT, and PET images. In this study, one patient was randomly selected from each MRI sequence (T1, T2, PD, FLAIR, and DWI) to construct the training dataset. The testing dataset consisted of 16 FLAIR MRI volumes and 30 MRI volumes from each of the T1, T2, PD, and DWI sequences.

To vary the training dataset size for a given patient’s MRI, we employ a controlled sampling technique with different stride sizes to approximate the few-shot setting with limited data. For each MRI sequence, we randomly select a single subject’s MRI volume (scan with multiple slices) and extract 2D slices along the acquisition axis. Note that if the MRI volume is a 3D acquisition, we resize it by the voxel-size fraction before extracting 2D slices along all three dimensions (sagittal, axial, and coronal). Instead of using all available slices, we sample every *k-th* slice along the acquisition dimensions, where *k* is the stride used. This simulates real-world constraints in which only sparse annotations or limited slices are available per patient, especially when MRI is acquired with a large slice thickness. For example, a stride of 5 doubles the number of training slices compared to a stride of 10, allowing us to regulate the size of the training dataset in a systematic manner.

##### 2.1.2.3 Multi-class anatomical segmentation dataset

For the multi-class anatomical segmentation task, we build datasets that include both hand-annotated segmentation masks and software-generated results as training targets. The hand-annotated cohort is MRBrainS18, and the software results are from NACC with FreeSurfer-generated annotations. The cohort’s details and methods used to build the fine-tuning datasets are introduced below.

###### 2.1.2.3.1 MRBrainS18

MRBrainS18 is part of the MR Brain Segmentation Challenge 2018, organized during the MICCAI 2018 conference. It is a public benchmarking dataset and competition platform designed to evaluate algorithms for multi-structural brain segmentation on 3 T MRI scans, especially in the presence of large brain pathologies. In this study, we select only T1 MRI scans from 7 subjects to form the training dataset and 23 subjects to form the testing dataset. The anatomical segmentation focuses on 8 regions, excluding the hyperintensity class.

###### 2.1.2.3.2 NACC

When using FreeSurfer to generate annotated masks for NACC, we choose 13 commonly observed brain regions as targets. All samples used for annotation are restricted to T1 sequences, as such modality often yields the most accurate and stable results in both hand annotations and software-generated outputs. For MRBrainS18 and NACC we sample only T1 3D MRIs as volumes instead of randomly pulling 2D slices from MRI scans. In each volume, we remap the 3D MRI to 2D slices along three axes: sagittal, axial, and coronal, and filter out MRI slices with no meaningful mask (most are slices with no brain tissue). The NACC dataset is used to perform tests with controlled dataset sizes by randomly sampling volumes from 10 to 100 and corresponding patients from 6 to 63. The test dataset built for software annotation includes fixed 2,434 volumes and 1,190 patients from NACC. All T1 MRI scans (approximately 440,000 slices) used for both training and testing have been pre-excluded from pretraining datasets. However, because exclusion is performed at the scan level rather than the patient level, there remains a possibility that non-T1 scans from a given patient appear during pretraining while T1 scans from the same patient are used during fine-tuning. Since the pretraining and fine-tuning scans are unregistered (i.e., not spatially aligned), were acquired under different protocols, and were used only for non-pathological tasks, we assume that cross-sequence correlation is negligible and does not constitute meaningful data leakage.

### 2.2 MAE pretraining

**Figure 2:**
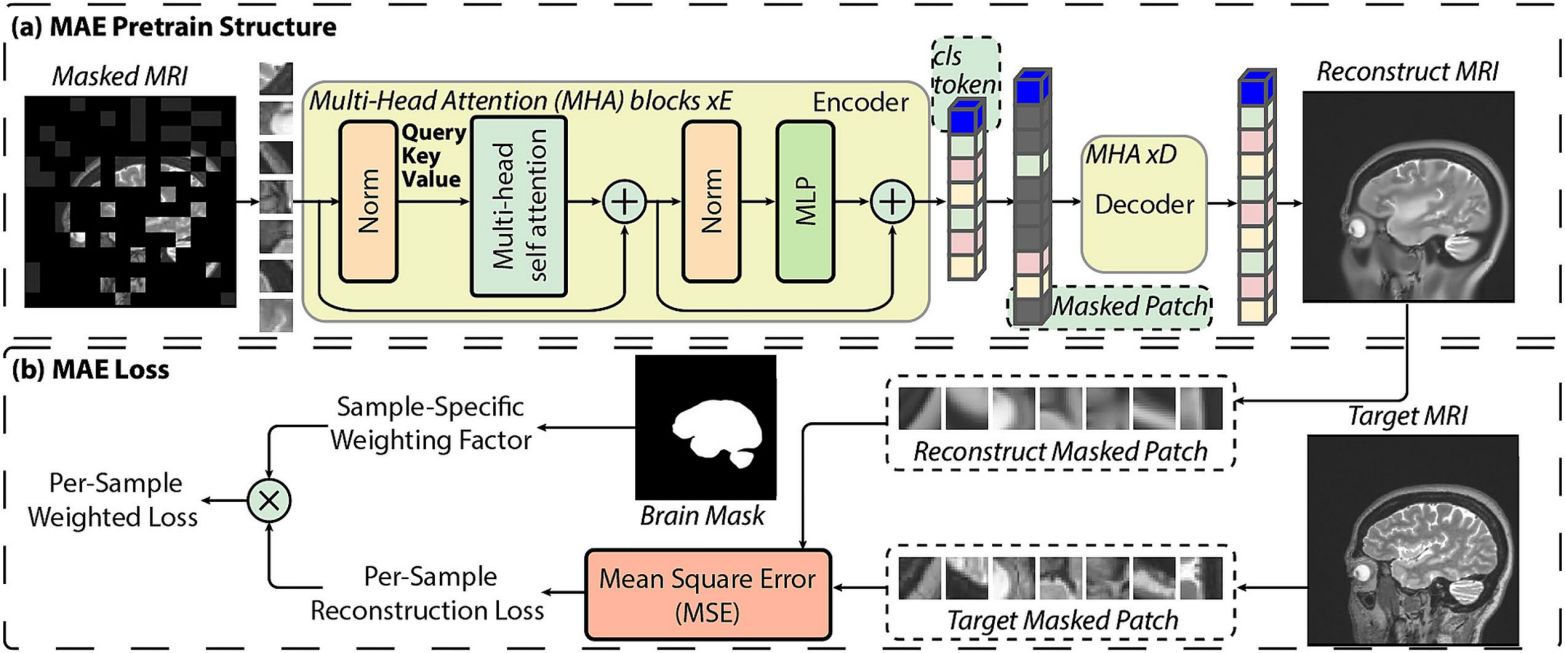
**(a)** Overview of the proposed MAE pretraining structure for brain MRI. MRI patches are encoded via a transformer encoder with *E* Multi-Head Attention (MHA) blocks, followed by a lightweight decoder (*D* MHA blocks) that reconstructs only the masked regions. **(b)** Illustration of the sample-specific loss computation. Mean squared error is calculated on reconstructed masked patches, and the per-sample loss is adaptively weighted based on brain-region coverage derived from a binary brain mask.

The MAE pretraining implemented in this study builds on the original Masked Autoencoder framework by adjusting it specifically for brain MRI datasets. Similar to the original MAE, the model consists of a large transformer encoder and a lightweight decoder, with the target set to reconstruct masked image patches. The details of the model structure are demonstrated in Figure 2a. The original MAE computes the reconstruction loss over the masked patches, while our loss function also introduces a sample-wise weighting scheme to address the partial-brain coverage that commonly occurs in aggregated MRI datasets. Using brain masks generated with FreeSurfer as reference (Dale and Sereno, 1993; Dale et al., 1999; Fischl et al., 1999a,b; Fischl and Dale, 2000; Fischl et al., 2001; Fischl et al., 2002; Kuperberg et al., 2003; Fischl et al., 2004a,b; Desikan et al., 2006; Han et al., 2006; Jovicich et al., 2006; Reuter et al., 2012), scans containing limited brain tissue, such as those dominated by non-brain regions or cropped fields of view, are assigned lower weights during training. The pipeline for sample-specific weighted loss is illustrated in Figure 2b. For the entire batch with *N* samples, we compute the final loss as the weighted average of the per-sample losses ℒ :


$$L=\frac{1}{N}\sum_{i=1}^{N}w_{i}{\tilde{l}}_{i}$$ (1)

Where ${\tilde{l}}_{i}$ is the per-sample loss, averaged over masked patches; $w_{i}$ is a sample-specific weighting factor based on the brain area coverage of the sample i .

From an optimization perspective, the sample-weighted MAE objective can be interpreted by decomposing the per-slice reconstruction loss according to brain-tissue and non-brain contributions. In this case, the per-sample loss ${\tilde{l}}_{i}$ denotes the reconstruction loss of the i -th 2D MRI slice computed over masked patches. Due to heterogeneous acquisition protocols and field-of-view, individual slices may exhibit varying degrees of brain coverage. We model the per-sample loss as:


$${\tilde{l}}_{i}=\alpha_{i}l_{i}^{\text{brain}}+(1-\alpha_{i})l_{i}^{\text{nonbrain}}$$ (2)

Where $\alpha_{i}\in [0,1]$ denotes the fraction of brain-tissue area estimated from a binary brain mask. When brain coverage is limited (i.e., small $\alpha_{i}$ ), the reconstruction loss is dominated by background regions or field-of-view artifacts that are weakly correlated with neuroanatomical structure and downstream brain imaging tasks. To account for this imbalance, each sample is assigned a weight $w_{i}=f(\alpha_{i})$ , where f(·) is a monotone increasing function of brain coverage. The resulting batch gradient with respect to model parameters θ is given by:


$$\begin{array}{c} \nabla_{\theta }L=\frac{1}{N}\sum_{i=1}^{N}w_{i}\nabla_{\theta }{\tilde{l}}_{i} \end{array}$$ (3)

This formulation aims to reduce the influence of slices with limited informative brain content while emphasizing samples that contain meaningful anatomical structure. Consequently, optimization is biased toward minimizing reconstruction error over brain-relevant regions rather than background-dominated areas. This interpretation suggests that the proposed weighting scheme functions (Equations 1–3). as an importance-weighting mechanism that aligns the MAE pretraining objective with the effective data distribution of interest for downstream neuroimaging tasks. This provides a principled motivation for its use when pretraining on large, heterogeneous MRI datasets aggregated across cohorts and acquisition protocols.

During pretraining, all 2D slices are preprocessed into the shape of 224×224 with a 0.1 to 99.9% pixel value clamp and max-min normalization. Data augmentation includes random rotation, flipping, and cropping. The pretrained model size is set to be the same as ViT-Base from the original MAE paper (He et al., 2022), with a 16×16 patch size and 12 transformer layers in the encoder. More detailed mathematical work and training implementation can be found in Supplementary Information S1.

### 2.3 Direct classification

To assess the utilization of pretrained transformer encoders in MAE for straightforward classification tasks such as sequence detection, we implemented a direct classification strategy using only the pretrained classification [CLS] token. Our approach repurposes the MAE transformer as a fixed feature extractor when adapting to downstream tasks. During fine-tuning, the encoder weights are frozen to preserve the learned representations, leading to fast downstream adaptations with fewer trainable parameters.

**Figure 3:**
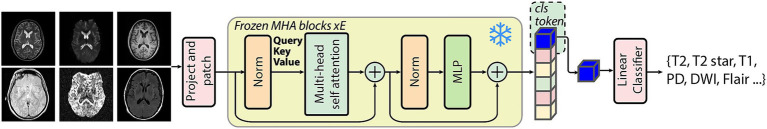
Direct classification pipeline for MRI sequence detection. Input MRI slices from multiple sequences are first tokenized via patch embedding and passed through the pretrained MAE encoder, consisting of *E* frozen multi-head attention blocks. A [CLS] token summarizes the global representation, which is then fed into a lightweight linear classifier to predict the MRI sequence label (e.g., T1, T2, FLAIR, DWI, PD, T2*).

As illustrated in Figure 3, input MRI slices from various image modalities, such as T1, T2, FLAIR, DWI, PD, and T2*, are first tokenized using a patch embedding projection layer. Then the patched tokens are processed through the frozen MAE encoder, comprising multiple stacked multi-head attention (MHA) and feedforward layers. The final [CLS] token embedding encodes the global context of the input image and is used as input to a lightweight linear classifier. The classifier is trained to predict the MRI sequence label using the AdamW optimizer (Loshchilov and Hutter, 2017) and $1\times 10^{-4}$ learning rate. For a multi-class logits classification task, such as sequence detection, the loss function is set to the cross-entropy loss:


$$\begin{array}{c} \;L_{\mathrm{ce}}=-\log(\frac{e^{z_{y}}}{\sum_{j=1}^{C}e^{z_{j}}})=-z_{y}+\log(\sum_{j=1}^{C}e^{z_{j}}) \end{array}$$ (4)

Where $z_{j}$ is the raw logit for class j , y is the correct class index, and C is the number of classes.

Within a representation learning framework, the above linear probing scheme admits a straightforward theoretical interpretation in the few-shot regime. Let x denote an input MRI slice and the class index y∈{1,,…,,C} denote the corresponding sequence label. A pretrained MAE encoder defines a feature mapping $\phi (x)\in {\mathbb{R}}^{d}$ . In MAE-classify, the encoder is kept frozen and a linear classifier $W\in {\mathbb{R}}^{C\times d}$ is trained by minimizing the cross-entropy loss:


$$\begin{array}{c} \;\min_{W}\frac{1}{n}\sum_{i=1}^{n}L_{\mathrm{ce}}(\mathrm{W\phi}(x),y_{i}) \end{array}$$ (5)

Compared with end-to-end fine-tuning of a high-capacity neural network, this formulation (Equations 4, 5) restricts the hypothesis class to linear predictors defined over a fixed representation space. In learning-theoretic terms, the generalization behavior of linear classifiers is governed primarily by the dimensionality, norm, and class separability (e.g., margin) of the learned features, rather than by the parameter count of the frozen backbone. Freezing the MAE encoder therefore substantially reduces effective model capacity, thereby mitigating overfitting in data-limited settings.

Under the frozen-encoder linear probing formulation, MAE pretraining learns a representation ϕ(x) that organizes MRI slices according to global semantic attributes such as acquisition sequence, contrast mechanism, and signal distribution. The downstream linear classifier then requires only minimal supervision to separate classes within this representation space. In short, the proposed linear probing setup serves two critical purposes: (1) evaluating the discriminative quality of the learned representations from MAE pretraining, and (2) demonstrating the few-shot adaptability of the frozen MAE transformers to classification tasks with minimal parameter overhead. Since the inputs are random, minimally preprocessed MRI slices with no modality-specific preprocessing, filtering, or handcrafted feature engineering, this pipeline is broadly applicable to heterogeneous real-world MRI datasets.

### 2.4 Segmentation with fused embedding

#### 2.4.1 MAE fusion architecture

**Figure 4:**
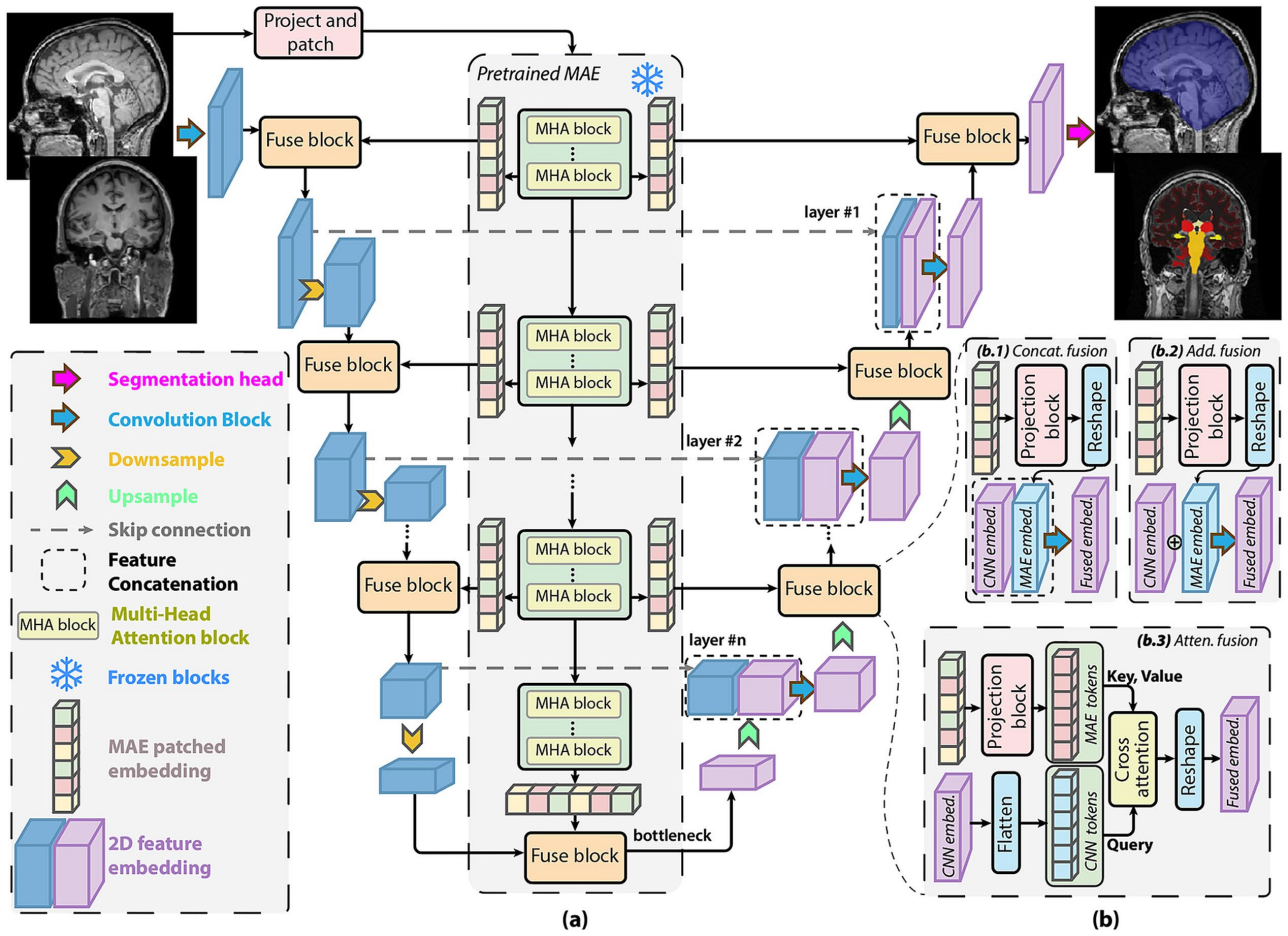
MAE-FUnet architecture for multi-class brain segmentation. **(a)** Overall structure fuses hierarchical CNN features with frozen MAE transformer embeddings through progressive decoder stages, incorporating skip connections and multi-scale feature integration. **(b)** Three possible fusion strategies are explored: **(b.1)** Concatenation-based fusion, **(b.2)** addition-based fusion, and **(b.3)** cross-attention-based fusion between CNN and transformer features.

To effectively adapt the pretrained MAE backbone for pixel-level tasks such as skull stripping and anatomical segmentation, we design a hybrid encoder-decoder architecture that fuses hierarchical CNN features with pretrained MAE transformer embeddings. This MAE-fused U-Net structure is referred to as the MAE-FUnet. As shown in Figure 4a, input MRI slices are first tokenized into patches and passed through the pretrained MAE transformer with frozen weights. In parallel, the same images are processed through a CNN-based U-Net backbone to extract multi-scale spatial features. The training configuration is set with an AdamW optimizer, a $1\times 10^{-4}$ learning rate, and a batch size of 48.

To integrate global representation from the transformer layers with local CNN features, we introduced a series of fusion blocks throughout multiple decoder levels. In total, three distinct fusion strategies are explored, as illustrated in Figure 4b: concatenation, addition, and cross-attention.

- *(b.1) Concatenation fusion:* Features from the CNN and MAE streams are first projected and reshaped to compatible dimensions and then concatenated along the channel dimension.
- *(b.2) Additive fusion:* MAE embeddings and CNN features are aligned in shape and dimension and then combined through element-wise summation.
- *(b.3) Attention fusion:* The CNN features are treated as query tokens and fused with MAE embeddings (key, value tokens) using a lightweight cross-attention module, allowing dynamic weighting of global context.

Same as the U-Net encoder-decoder structure, skip connections preserve resolution details from earlier stages, and upsampling layers progressively restore spatial dimensions. The final convolutional segmentation head outputs dense pixel-wise predictions for brain tissues and anatomical structures. Notably, the MAE weights remain frozen to ensure stable generalization under few-shot conditions, thereby reducing the trainable parameter count.

In the context of dense prediction, the MAE-FUnet fusion design aims to infer a pixel-wise label field y from an input image x under few-shot supervision. Convolutional encoders produce hierarchical feature maps $f_{s}(x)$ at multiple spatial scales s , which are well-suited for capturing local textures, edges, and boundary information. In contrast, transformer embeddings g(x) derived from MAE pretraining encodes long-range dependencies and global anatomical context but lacks explicit spatial inductive bias. In MAE-FUnet, fusion is performed by combining CNN features with projected transformer embeddings:


$$\begin{array}{c} \;h_{s}(x)=\text{Fuse}(f_{s}(x),\Pi_{s}(g(x))) \end{array}$$ (6)

Where $\Pi_{s}(\cdot )$ denotes a projection operator that aligns transformer embeddings with the spatial resolution and channel dimensionality at scale s . This fusion (Equation 6) augments local feature representations with global contextual information, improving class separability for anatomically ambiguous or small structures that are difficult to resolve using local cues alone.

From a bias–variance perspective, different fusion mechanisms introduce different levels of interaction complexity. Concatenation-based fusion preserves complementary information from both streams while maintaining a relatively simple aggregation structure. In contrast, attention-based fusion introduces additional adaptive interactions and trainable parameters, increasing model flexibility but also amplifying estimation variance when supervision is limited. As a result, attention-based fusion may exhibit reduced stability in few-shot regimes despite its higher expressive power. This theoretical consideration aligns with the empirical findings (further elaborated in Sections 3.2–3.4), where MAE-FUnet with concatenation fusion achieves the best trade-off between accuracy and robustness, demonstrating lower performance variance across different training sample sizes and datasets.

#### 2.4.2 Hybrid loss function

To achieve competitive performance on multiple brain regions segmentation, especially when handling class imbalance caused by the rarity of certain regions due to size and anatomical location, we introduce a composite loss function that integrates *Dice loss*, *Focal loss*, and *Cross-Entropy loss* (Equations 7–10). Each component addresses distinct challenges in medical segmentation, including class imbalance, partial-volume effects, and small-structure delineation. The final loss is a weighted sum of the three terms:


$$\begin{array}{c} \;L_{\text{total}}=L_{\text{dice}}+L_{\text{focal}}+L_{\mathrm{ce}} \end{array}$$ (7)

The detailed mathematical formulation of each loss term is provided below.

*Dice Loss:* We use a non-batch variant of the soft Dice loss computed per sample, including the background class (unmasked area). Let $p_{i,c}$ and $g_{i,c}$ be the softmax prediction and the ground truth indicator for the pixel i and class c , then:


$$\begin{array}{c} L_{\text{dice}}=1-\frac{2\sum_{i}p_{i,c}g_{i,c}+\in }{\sum_{i}p_{i,c}^{2}+\sum_{i}g_{i,c}^{2}+\in } \end{array}$$ (8)

Where $\in =10^{-5}$ is a smoothing constant to prevent division by zero.

*Focal Loss:* To mitigate class imbalance, we employ the standard focal loss formulation:


$$\begin{array}{c} L_{\text{focal}}=-\alpha {\left(1-p_{i,c}\right)}^{\gamma }\log(p_{i,c}) \end{array}$$ (9)

Where γ = 2.0, α = 0.25, and $p_{i,c}$ denotes the predicted probability of the true class. This loss down-weights well-classified (easy) pixels and emphasizes harder, misclassified pixels during training, thereby improving learning on underrepresented and challenging brain regions.

*Cross-Entropy Loss:* We include the standard pixel-wise multi-class cross-entropy loss:


$$\begin{array}{c} L_{\mathrm{ce}}=-\sum_{c}g_{i,c}\log(p_{i,c}) \end{array}$$ (10)

This term enforces accurate pixel-level classification and complements the Dice and Focal components to improve convergence.

### 2.5 Evaluation metrics

#### 2.5.1 Sequence detection metrics

The performance of the sequence detection task is evaluated using accuracy, precision, and recall, together with three complementary variants of the F1 scores: micro-, macro-, and weighted-averaged F1 (Equations 11–17). Each metric captures a different aspect of model behavior. The micro F_1_ score treats every sample equally and is dominated by the most frequent class. Meanwhile, the macro F1 score assigns equal weight to each class, making it insensitive to class frequency and more reflective of performance on underrepresented sequences, therefore addressing class imbalances. The Weighted F_1_ score weighs each class metric by its size, thereby penalizing bad performance on common classes while still reflecting class imbalance. The corresponding equations of precision, recall, and the three F1 scores are defined as follows:

With class k∈{1,,…,,K} , $TP_{k}$ as true positives, $FP_{k}$ as false positives, $FN_{k}$ as false negatives, $TN_{k}$ as true negatives, $N_{k}=TP_{k}+FN_{k}$ as the number of ground-truth samples of class k .

Total number of samples:


$$\begin{array}{c} N=\sum_{k=1}^{K}N_{k} \end{array}$$ (11)

Overall accuracy:


$$\begin{array}{c} \text{Accuracy}=\sum_{k=1}^{K}TP_{k} \end{array}$$ (12)

Overall precision:


$$\begin{array}{c} \text{Precision}=\frac{\sum_{k=1}^{K}TP_{k}}{\sum_{k=1}^{K}(TP_{k}+FP_{k})} \end{array}$$ (13)

Overall recall:


$$\begin{array}{c} \text{Recall}=\frac{\sum_{k=1}^{K}TP_{k}}{\sum_{k=1}^{K}(TP_{k}+FN_{k})} \end{array}$$ (14)

Micro-averaged F_1_ score:


$$\begin{array}{c} F_{1\text{micro}}=\frac{2\sum_{k=1}^{K}TP_{k}}{2\sum_{k=1}^{K}TP_{k}+\sum_{k=1}^{K}FP_{k}+\sum_{k=1}^{K}FN_{k}} \end{array}$$ (15)

Macro-averaged F_1_ score:


$$\begin{array}{c} F_{1\text{macro}}=\frac{1}{K}\sum_{k=1}^{K}\frac{2TP_{k}}{2TP_{k}+FP_{k}+FN_{k}} \end{array}$$ (16)

Weighted-averaged F_1_ score:


$$\begin{array}{c} F_{1\text{weighted}}=\sum_{k=1}^{K}\frac{N_{k}}{N}(\frac{2TP_{k}}{2TP_{k}+FP_{k}+FN_{k}}) \end{array}$$ (17)

#### 2.5.2 Skull stripping and anatomical segmentation metrics

To quantitatively evaluate the performance of our MAE-FUnet on segmentation tasks, we adopt two commonly used evaluation metrics in medical image segmentation: the Dice score and Intersection over Union (IoU) (Equations 18, 19). These two metrics are often used to evaluate performance on imbalanced and multi-class anatomical structures, as they provide meaningful measures of overlap between predicted and ground-truth segmentations.

*Dice Score:* The Dice Score, also known as the F1 score in binary segmentation, is defined as:


$$\begin{array}{c} \text{Dice}=2\times \frac{|P\cap G|}{|P|+|G|} \end{array}$$ (18)

Where P is the set of predicted positive pixels and G is the set of ground-truth positive pixels.

*Intersection over Union (IoU):* The Intersection over Union, also referred to as the Jaccard Index, is defined as:


$$\begin{array}{c} \mathrm{IoU}=\frac{|P\cap G|}{|P\cup G|} \end{array}$$ (19)

Where P and G are the same as in Dice score (Equation 18).

## 3 Results

If not explicitly indicated, all experiments are conducted using a consistent training configuration, including the AdamW optimizer with a learning rate of $1\times 10^{-4}$ and a batch size of 48. The data preprocessing involves standard max-min normalization with 0.1–99.9% clamping, followed by data augmentation using random flipping, rotation, and center cropping.

### 3.1 Sequence detection

To evaluate the classification capability of pretrained MRI transformers, we deploy a widely studied medical image classification task: MRI sequence detection (Mahmutoglu et al., 2023; Helm et al., 2024; Ali, 2024; Pan et al., 2025). To tackle this task under few-shot conditions, we construct the training dataset comprising MRIs across seven MRI sequence types: T1, T2, FLAIR, PD, T2* (Gaillard et al., 2025), Susceptibility-Weighted Imaging (SWI) (Tang et al., 2014), and DTI/DWI. As detailed in section 2.1.2.1, for each sequence, we randomly sample *n* slices from three datasets: NACC, OASIS, and ADNI. By procedurally increasing *n* from 10 to 100, we simulate the few-shot conditions across varying dataset sizes. For comparison, we benchmark our method, *MAE-classify* (introduced in Section 2.3), against established architectures including U-Net, ResNet, EfficientNetv2, and MedViT. As shown in Tables 3, 4, our lightweight MAE-based classifier consistently outperforms strong baselines with an overall accuracy of 99.24%, precision of 99.19%, and recall of 99.16%. In addition, the MAE-classify achieves the highest micro, macro, and weighted F1 scores simultaneously, suggesting that MAE-classify not only delivers high overall accuracy but also maintains balanced and robust classification performance without being biased by class imbalance.

**Table 3:** Overall MRI sequence classification performance, including accuracy, precision, recall, and F1 score, for the proposed MAE-classify model and baseline methods, along with their numbers of trainable parameters (in millions, M).

Method	Accuracy (%)	Precision (%)	Recall (%)	*F*_1_ Score (%)			Trainable params
				Micro	Macro	Weighted	
U-Net	93.42	93.45	93.45	93.45	93.34	93.43	32 M
EfficientNetV2	98.59	98.59	98.59	98.59	98.53	98.59	20 M
ResNet	98.10	98.07	98.04	98.11	98.05	98.11	11 M
MedViT	95.05	95.16	95.01	95.05	95.05	95.05	31 M
*MAE-classify*	**99.24**	**99.19**	**99.16**	**99.23**	**99.17**	**99.23**	**6,152**

F1 scores are reported using micro, macro, and weighted averaging. All models are trained on a fine-tuning subset containing 30 slices per sequence per dataset. Bold values indicate the best performance among all compared methods for each metric.

**Table 4:** MRI sequence classification accuracy for the proposed MAE-classify model and baseline methods.

Method	Accuracy over sequence (%)						
	T1	T2	FLAIR	PD	T2*	SWI	DTI/DWI
U-Net	94.62	94.19	86.12	89.94	96.63	95.30	97.06
EfficientNetV2	98.83	97.85	98.71	96.82	98.64	98.93	99.87
ResNet	98.09	98.39	97.17	95.66	98.61	98.34	**99.90**
MedViT	94.94	95.17	94.85	91.83	97.16	92.28	98.82
*MAE-classify*	**99.51**	**99.43**	**99.64**	**97.39**	**99.42**	**99.31**	99.51

Accuracy (%) is reported separately for each MRI sequence. All models are trained on a fine-tuning subset consisting of 30 slices per sequence per dataset. Bold values indicate the best performance among all compared methods for each metric.

**Figure 5:**
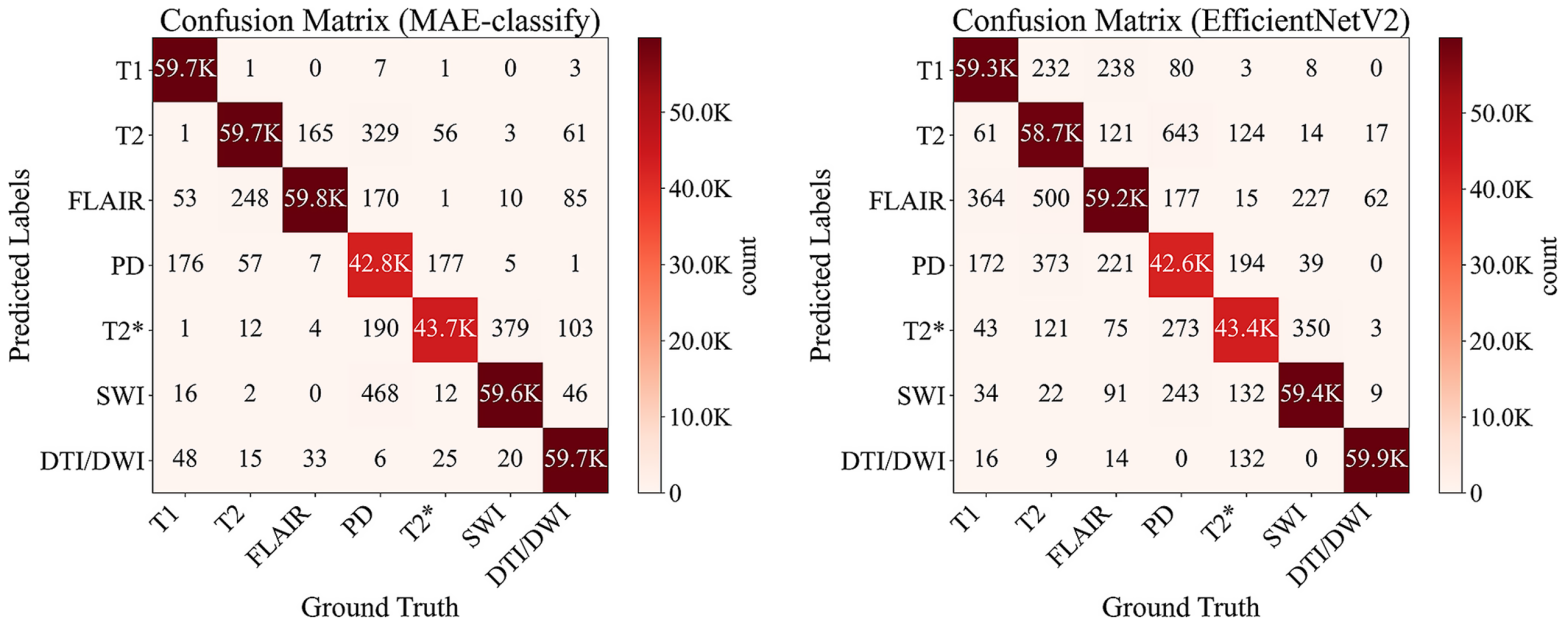
Confusion matrices for MRI sequence classification on the test dataset, comparing the MAE-based classifier (left) and EfficientNetV2 (right), both trained on 30 slices/sequence/dataset. Rows denote predicted sequence labels and columns denote ground-truth labels. The diagonal entries represent correct classifications, while off-diagonal entries indicate misclassifications across MRI sequences. Color intensity reflects the number of slices.

Specifically, in Table 4 with models trained on 30 slices per sequence per dataset, MAE-classify achieved the highest classification accuracy in six out of seven categories, including T1 (99.51%), T2 (99.43%), FLAIR (99.64%), PD (97.39%), *T2 Hemo (99.42%), and *T2 SWI (99.31%). Even in the DTI/DWI category, where ResNet slightly surpasses it with 99.90%, MAE-classify still maintains a strong performance at 99.51%. Compared with the most competitive baseline EfficientNetV2 (second-highest overall accuracy), the MAE-classify confusion matrix in Figure 5 shows a noticeably stronger diagonal dominance and fewer off-diagonal errors across most MRI sequences. In particular, confusions between visually similar sequences (e.g., T2 vs. PD or T1 vs. FLAIR) are substantially reduced, indicating more accurate and consistent predictions. These evaluations highlight the pretrained transformers’ ability to encode global contextual features that are critical for distinguishing fine-grained sequence characteristics. Additionally, MAE-classify achieved this with only 6,152 trainable parameters, whereas other models often take up 11–32 million trainable parameters. Such a comparable size difference demonstrates the efficiency of self-supervised pretraining and minimal task-specific tuning, especially when encountering direct classification such as modality detection.

**Figure 6:**
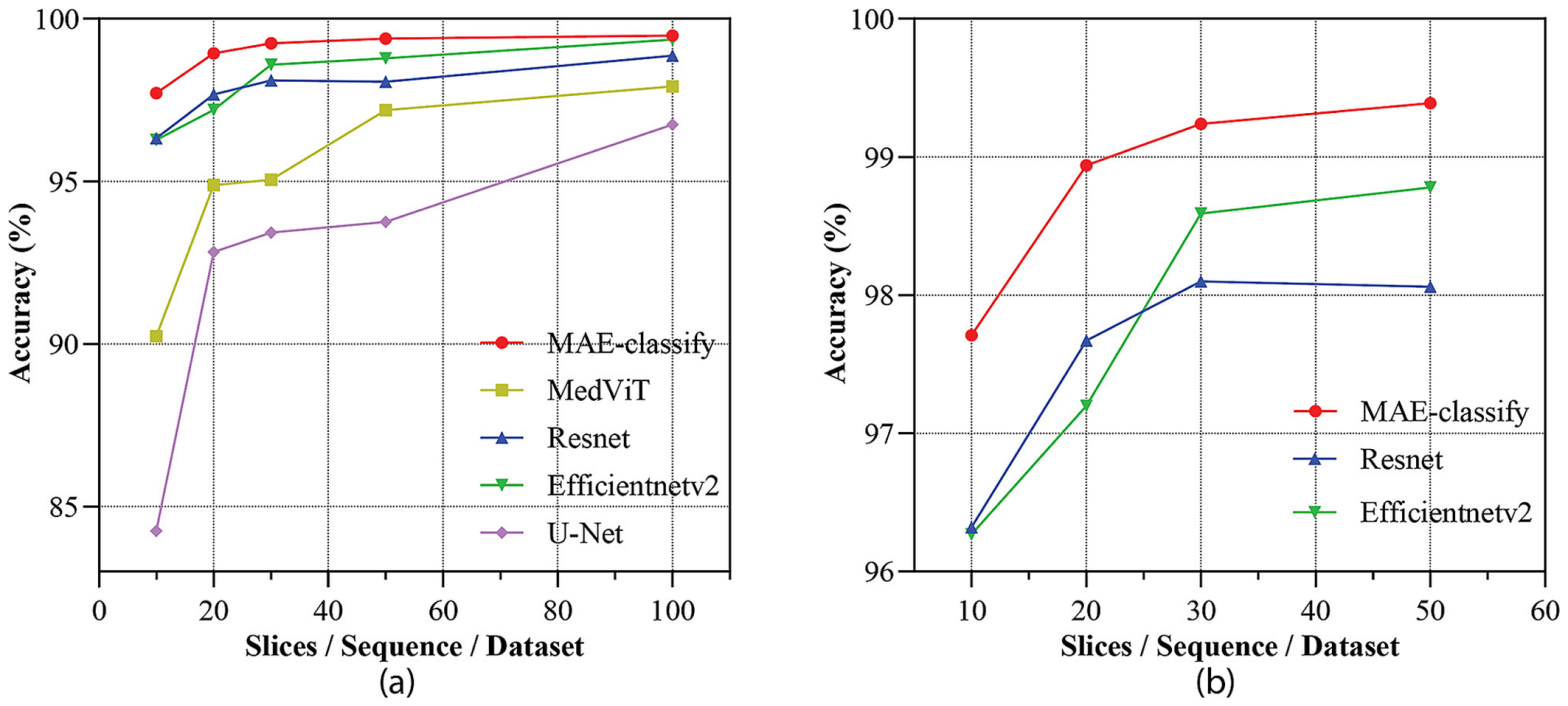
Performance of MRI sequence classification under varying sample sizes: **(a)** Classification accuracy across 7 MRI modalities with the number of slices increasing from 10 to 100 and **(b)** zoom in performance on MAE-classify, ResNet, and EfficientNetV2 with limited slices from 10 to 50.

We further examine sample efficiency by conducting few-shot evaluations in which the number of training slices per sequence is systematically varied. As illustrated in Figure 6, although all models benefit from increased training data, MAE-classify outperforms the other models at comparable training set sizes. MAE-classify approaches 98.9% accuracy with just 20 slices, while competitive baselines such as ResNet and EfficientNetV2, require larger training sets to reach comparable accuracy. Meanwhile, U-Net and MedViT converge slowly and tend to plateau at lower accuracies, even when trained with up to 100 slices. These observations confirm the robustness and data efficiency of MAE-classify in few-shot scenarios, which is important in medical practice where annotated data is often limited.

### 3.2 Skull stripping

To evaluate the segmentation performance of the proposed MAE-FUnet architecture for skull stripping, we compare it against multiple baseline models across several benchmark datasets. The comparison models consist of widely used architectures designed for medical image segmentation. The selected baselines represent diverse architectural paradigms, including CNN-based, transformer-based, and hybrid models. U-Net serves as a pure CNN-based structure that uses the same backbone configuration as the CNN component in MAE-FUnet. SegFormer is a pure transformer-based architecture with multi-scale skip connections. Swin-Unet is also transformer-based and constructed as a U-Net architecture, but with the Swin Transformer (Liu et al., 2021) replacing CNN modules. TransUNet is a hybrid architecture that combines both transformer and U-Net structures, with transformer modules appended only at the bottleneck layer of the U-Net. The MAE-direct model follows a similar design to the MAE-based classifier shown in Figure 3, but replaces the classification head with a multi-layer CNN segmentation head, without incorporating feature fusion.

In Table 5, we report Dice and IoU scores on two benchmark brain MRI datasets, NFBS and SynthStrip. The stride size is fixed to 7 for all datasets and sequence modalities. The proposed MAE-FUnet used in this experiment comprises four downsampling/upsampling layers and a bottleneck layer, with the first-layer channels set to 64. The corresponding MAE-pretrained transformer layers selected for fusion are the 1st, 3rd, 6th, 9th, and 12th layers of its encoder. The fusion strategy is set to concatenation. MAE-FUnet achieves the highest Dice and IoU scores across nearly all modalities on both datasets, demonstrating the effectiveness of fusing transformer embeddings with CNN features for skull stripping. Furthermore, the underperformance of MAE-direct compared to other baselines, along with the consistent improvements achieved by MAE-FUnet, highlights the importance of explicit feature fusion in leveraging MAE representations for segmentation.

**Table 5:** Skull stripping performance (IoU/Dice%) across NFBS and SynthStrip datasets (sample stride = 7).

Method	NFBS	SynthStrip				
	T1	T1	T2	FLAIR	PD	DWI
U-Net	92.96/96.35	91.38/95.23	94.00/96.89	91.67/95.65	93.99/96.87	88.49/93.82
SegFormer	94.21/97.02	93.67/96.68	93.97/96.88	93.26/96.51	94.04/96.92	90.89/95.18
TransUNet	94.13/96.97	90.05/94.35	91.98/95.76	92.98/96.36	93.69/96.72	89.76/94.52
Swin-Unet	90.33/94.92	81.15/88.93	81.56/89.07	80.99/89.24	87.34/92.97	75.21/85.10
MAE-direct	90.25/94.88	91.70/95.62	92.85/96.28	92.86/96.30	92.96/96.34	88.56/93.88
*MAE-FUnet*	**96.57/98.26**	**95.33/97.54**	**95.52/97.70**	**94.32/97.08**	**95.56/97.72**	**90.93/95.20**

Results are reported for five MRI sequences (T1, T2, FLAIR, PD, DWI) in SynthStrip and T1 in NFBS. Each value pair represents IoU/Dice. Bold values indicate the best performance among all compared methods for each metric.

**Figure 7:**
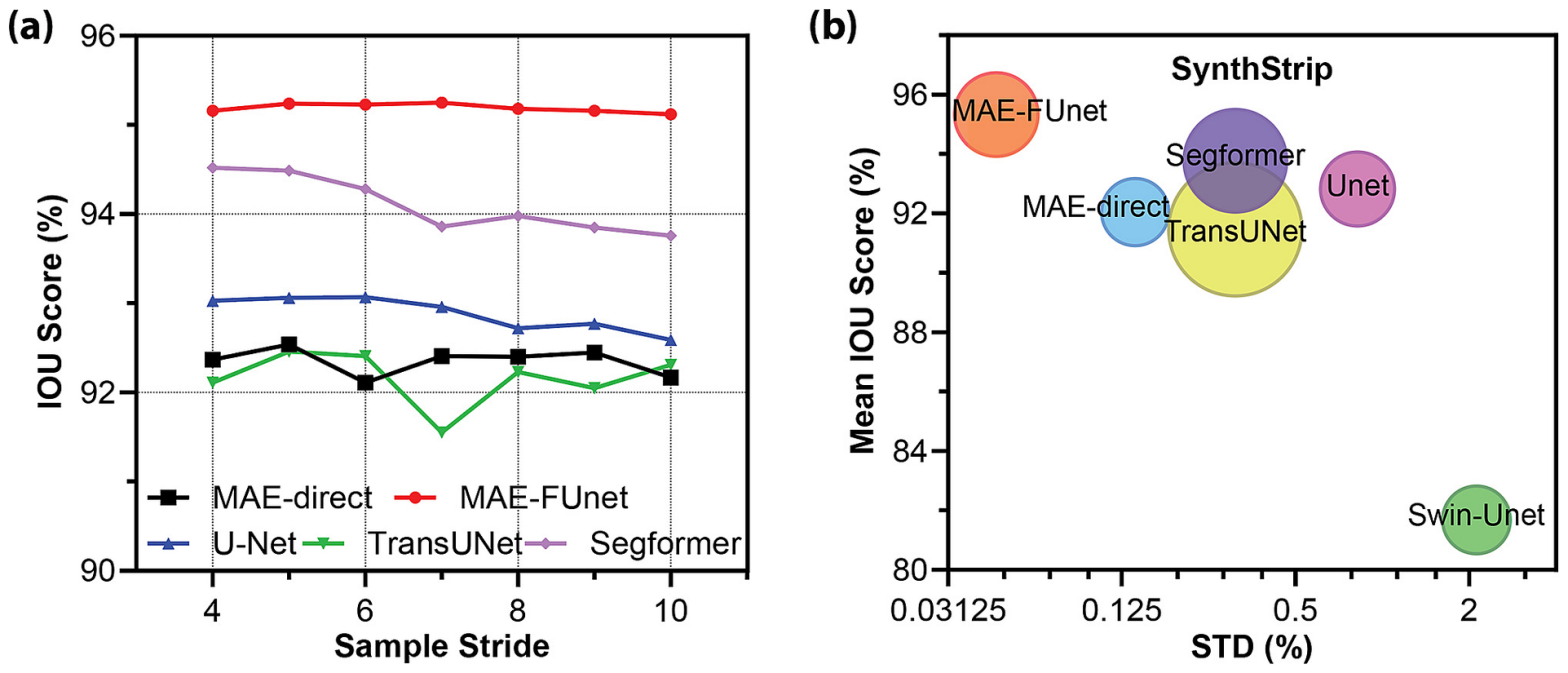
Performance of MRI skull stripping under varying sample sizes. **(a)** IoU score vs. sample stride on SynthStrip dataset. The line plot illustrates the segmentation performance trend as sample availability decreases. **(b)** Mean IoU vs. standard deviation (STD) for all models on SynthStrip. The bubble chart visualizes the trade-off between accuracy and robustness. Bubble size reflects trainable parameters across various baselines.

Robustness under sample scarcity is evaluated by varying the slice sampling stride from 4 to 10 on the SynthStrip dataset, which includes multiple MRI sequence types such as T1, T2, FLAIR, PD, and SWI. The performance comparison is reported in Table 6 as the mean Dice and IoU scores across all sequences and datasets. As expected, increasing the stride (i.e., reducing the training data) leads to a degradation in overall segmentation accuracy. However, MAE-FUnet outperforms others in stability, showing exceptional robustness with a mean IoU of 95.19% and the lowest standard deviation of 0.045%. This trend is also evident in Figure 7a, where MAE-FUnet consistently achieves higher performance and exhibits stability as the stride size increases. Figure 7b presents a bubble plot of mean IoU versus performance variance across all baselines, with bubble size indicating the number of trainable parameters. MAE-FUnet is positioned in the upper-left region of the plot, corresponding to high mean IoU and low variance, while maintaining a moderate model size. This indicates that MAE-FUnet achieves a favorable balance between segmentation accuracy, stability, and parameter efficiency. In contrast, models such as Swin-Unet exhibit lower mean IoU and higher variance, indicating reduced reliability under data-scarce conditions.

**Table 6:** Evaluation of SynthStrip with varying sample strides.

Sample stride	*MAE-FUnet*	MAE-direct	Swin-Unet	U-Net	TransUNet	SegFormer
4	**95.16/97.52**	92.37/96.03	81.69/89.89	93.03/96.38	92.11/95.89	94.52/97.18
5	**95.24/97.56**	92.54/96.12	83.50/90.99	93.06/96.40	92.46/96.08	94.49/97.16
6	**95.23/97.55**	92.11/95.89	83.87/91.21	93.07/96.41	92.41/96.05	94.28/97.05
7	**95.25/97.57**	92.41/96.05	82.06/90.12	92.96/96.35	91.55/95.58	93.86/96.83
8	**95.18/97.53**	92.40/96.05	82.58/90.44	92.72/96.22	92.23/95.96	93.98/96.90
9	**95.16/97.52**	92.45/96.08	83.61/91.05	92.77/96.25	92.05/95.86	93.85/96.83
10	**95.12/97.50**	92.17/95.92	77.76/87.45	92.59/96.15	92.31/96.00	93.76/96.78
Mean	**95.19/97.54**	92.35/96.02	82.15/90.16	92.89/96.31	92.16/95.92	94.11/96.96
STD (%)	**0.045/0.023**	0.142/0.077	1.949/1.200	0.176/0.094	0.284/0.156	0.295/0.154

Lower stride values correspond to denser slice sampling (more training samples), while higher strides reduce the training set size. Mean and standard deviation (STD) of IoU and Dice scores are provided to assess performance stability under limited data. Bold values indicate the best performance among all compared methods for each metric.

**Figure 8:**
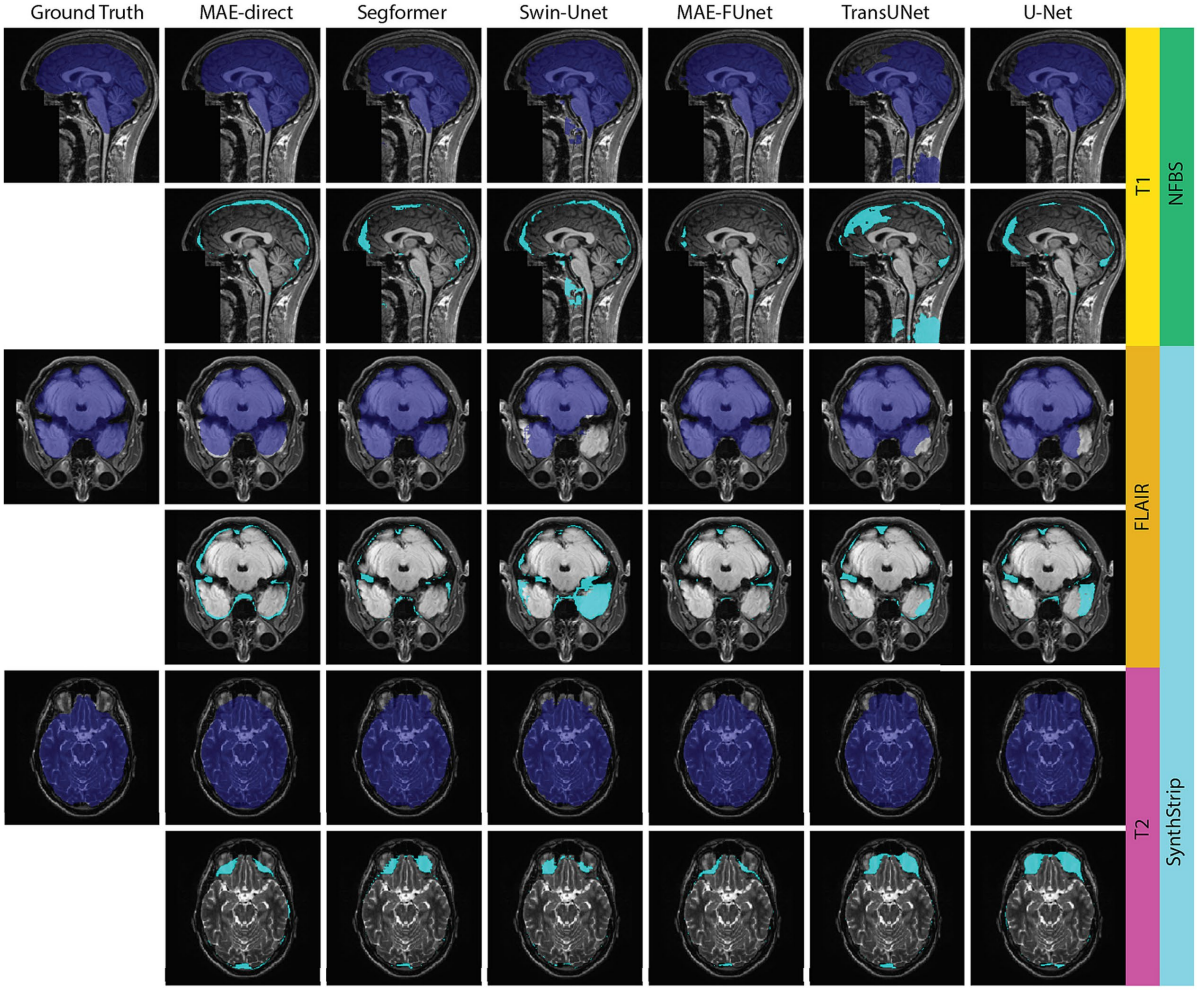
Visual comparison of skull stripping predictions across MRI sequences. Each row corresponds to a different MRI sequence (T1, T2, FLAIR for demonstration) from the NFBS and SynthStrip datasets. The top images show the predicted binary brain masks, and the bottom images display pixel-wise error maps (cyan) highlighting misclassified regions relative to ground truth.

We also include a qualitative visualization in Figure 8 to complement our quantitative results. Figure 8 presents representative skull stripping outcomes across multiple sequences from the NFBS and SynthStrip datasets. The comparison is made between the predicted brain masks (top of each row) and the corresponding error maps (bottom of each row), where cyan highlights mislabeled pixels. These examples demonstrate that the MAE-FUnet produces more anatomically consistent masks with fewer boundary errors. In contrast, several baseline models show consistent failure patterns. For example, TransUNet often includes non-brain structures, such as cervical spine tissue, within the predicted brain mask. Conversely, Swin-Unet often under-segments brain regions, notably missing portions of the anterior temporal lobes. In short, these results confirm that: (1) directly deploying pretrained MAE with appending modules, such as CNN heads, results in inferior segmentation, and (2) fusing hierarchical CNN features with MAE latent embeddings, as in MAE-FUnet, can substantially boost anatomical delineation in few-shot settings.

### 3.3 Multi-class anatomical segmentation

To further evaluate the performance of MAE-FUnet on more complex segmentation tasks, we conduct experiments for multi-class anatomical segmentation using two benchmark datasets: MRBrainS18 and NACC. The same model configuration used for skull stripping is applied here: MAE-FUnet with 4-layer depth, a first-layer dimension of 64, and a concatenation-based fusion strategy. By training on a dataset of 7 3D volumes for MRBrainS18 and 30 3D volumes for NACC, the results reported in Table 7 show that in both datasets, MAE-FUnet consistently outperforms baseline models, including U-Net, Swin-Unet, TransUNet, and SegFormer, across most brain regions. On MRBrainS18, it achieves the highest mean Dice score of 83.06% and IoU score of 72.41%, with notably strong performance on critical regions such as the cerebellum, ventricles, and brainstem. A similar trend is observed on NACC, where MAE-FUnet ranks first, with a mean Dice score of 83.70% and an IoU of 72.88%. In addition, the MAE-FUnet also demonstrates exceptional accuracy in segmenting small and anatomically complex regions. For example, MAE-FUnet outperforms the second-best baseline by +4.48% in Dice score for the hippocampus and by +4.90% for the amygdala. Meanwhile, MAE-FUnet achieves more modest improvements of 0.26 and 0.56% in relatively large regions such as the cerebral white matter and the cerebral cortex.

**Table 7:** Dice and IoU scores (%) for anatomical structure segmentation with 7 training samples on MRBrainS18 and 30 training samples on NACC datasets.

Region	IoU/Dice (%)				
	*MAE-FUnet*	Swin-Unet	U-Net	TransUNet	SegFormer
MRBrainS18					
Cortical gray matter	**71.64/83.48**	66.90/80.17	71.46/83.35	70.76/82.88	68.71/81.45
Basal ganglia	**68.81/81.48**	58.48/73.76	66.09/79.54	67.29/80.34	62.86/77.12
White matter	**76.56/86.72**	71.84/83.61	74.95/85.68	75.21/85.84	73.17/84.50
White matter lesions	40.10/57.01	27.24/42.47	37.84/54.68	**40.39/57.29**	35.03/51.80
Cerebrospinal fluid	**67.14/80.33**	60.84/75.64	66.69/80.01	65.45/79.10	65.06/78.82
Ventricles	**87.06/93.07**	81.16/89.59	85.72/92.31	85.47/92.14	80.56/89.21
Cerebellum	**87.16/93.14**	76.10/86.40	86.23/92.60	85.17/91.58	84.63/91.67
Brain stem	80.80/89.27	73.25/84.53	78.17/87.62	**81.90/89.89**	77.03/86.89
Mean	**72.41/83.06**	64.48/77.02	70.89/81.97	71.46/82.38	68.38/80.18
NACC					
Cerebral White Matter	**87.03/93.06**	83.71/91.13	86.44/92.72	85.70/92.29	86.58/92.80
Cerebral Cortex	**77.96/87.60**	72.06/83.74	76.46/86.65	74.47/85.35	77.40/87.25
Cerebellum White Matter	**75.91/86.11**	67.46/80.36	73.27/84.46	71.88/83.47	73.89/84.84
Cerebellum Cortex	**81.60/89.77**	73.11/84.35	79.26/88.36	77.25/87.06	78.98/88.17
Thalamus	**79.12/88.17**	69.99/82.15	78.01/87.54	74.04/84.92	74.52/85.26
Caudate	**75.04/85.54**	63.80/77.62	70.51/82.51	68.83/81.30	67.24/80.15
Putamen	**74.64/85.24**	62.20/76.44	71.62/83.28	70.92/82.76	65.06/78.54
Pallidum	**66.61/79.50**	51.64/67.59	63.51/77.40	63.31/77.18	58.83/73.60
Brainstem	**80.22/88.78**	71.84/83.39	77.69/87.30	77.72/87.26	77.11/86.87
Hippocampus	**73.24/84.30**	54.83/70.48	66.68/79.82	64.68/78.25	62.09/76.30
Amygdala	**64.79/78.14**	40.83/57.24	58.27/73.24	57.51/72.56	52.73/68.45
CSF	**57.71/72.40**	28.20/42.70	52.45/68.19	53.74/69.26	34.59/50.48
WM-hypointensities	**53.60/69.52**	41.69/58.52	50.21/66.69	47.47/64.12	45.88/62.68
Mean	**72.88/83.70**	60.10/73.52	69.57/81.40	68.27/80.44	65.76/78.11

Each dataset contains evaluation for separate regions as well as overall Dice/IoU mean scores across all regions. Bold values indicate the best performance among all compared methods for each metric.

**Figure 9:**
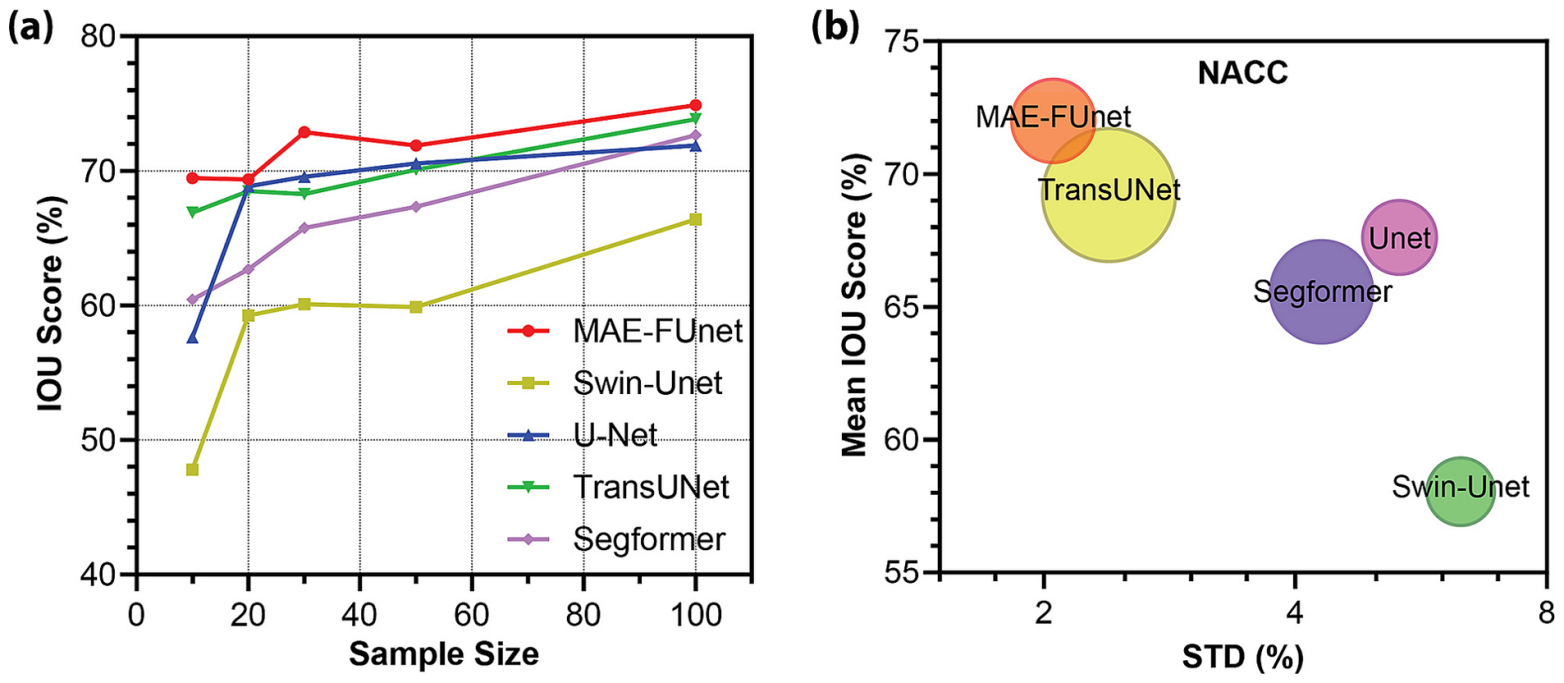
Performance of brain MRI multi-class segmentation under varying sample sizes. **(a)** IoU score trend across increasing sample sizes for few-shot anatomical segmentation on the NACC dataset. **(b)** Mean IoU vs. performance stability (STD) for all methods on the NACC dataset. Bubble size indicates the model’s trainable parameter count.

Moreover, we investigate robustness under data scarcity by simulating few-shot segmentation on the NACC dataset with training set sizes of 10, 20, 30, 50, and 100 MRI volumes, as summarized in Table 8. As expected, performance improves as the dataset size increases, but MAE-FUnet consistently outperforms competing methods across all sample sizes. Notably, it obtains an average Dice score of 82.89% and IoU of 71.70% across all volumes, outperforming all other models. It also exhibits the lowest variance, with standard deviations of 2.100% in IoU and 1.489% in Dice score, suggesting the best stability under different data-limited conditions. The trends are further visualized in Figures 9a,b. The line plot in Figure 9a highlights consistently higher IoU scores at all sample sizes for MAE-FUnet. In Figure 9b, MAE-FUnet is located in the upper-left region, corresponding to high mean IoU and low performance variability. When accounting for the number of trainable parameters, these results demonstrate that MAE-FUnet achieves strong accuracy and robustness with a relatively modest model size.

**Table 8:** Few-shot segmentation evaluation recorded as overall IoU and Dice score for all 13 regions on NACC.

Sample size	*MAE-FUnet*	Swin-Unet	U-Net	TransUNet	SegFormer
10	**69.45/81.31**	47.81/62.88	58.73/72.79	66.90/79.42	60.44/74.08
20	**69.37/81.25**	59.26/73.33	64.01/77.11	68.49/80.63	62.68/75.37
30	**72.88/83.70**	60.10/73.52	67.54/79.93	68.27/80.44	65.76/78.11
50	**71.89/83.03**	59.87/73.60	66.46/78.95	70.08/81.78	67.34/79.58
100	**74.88/85.17**	66.39/78.94	70.16/81.86	73.83/84.43	72.66/83.52
Mean	**71.70/82.89**	58.69/72.45	65.38/78.13	69.51/81.34	65.77/78.13
STD (%)	**2.100/1.489**	6.024/5.232	3.864/3.081	2.380/1.718	4.192/3.324

By varying sample size from 10 to 100, the mean and standard deviation (STD) of IoU and Dice scores are provided to assess performance stability under limited data. Bold values indicate the best performance among all compared methods for each metric.

**Figure 10:**
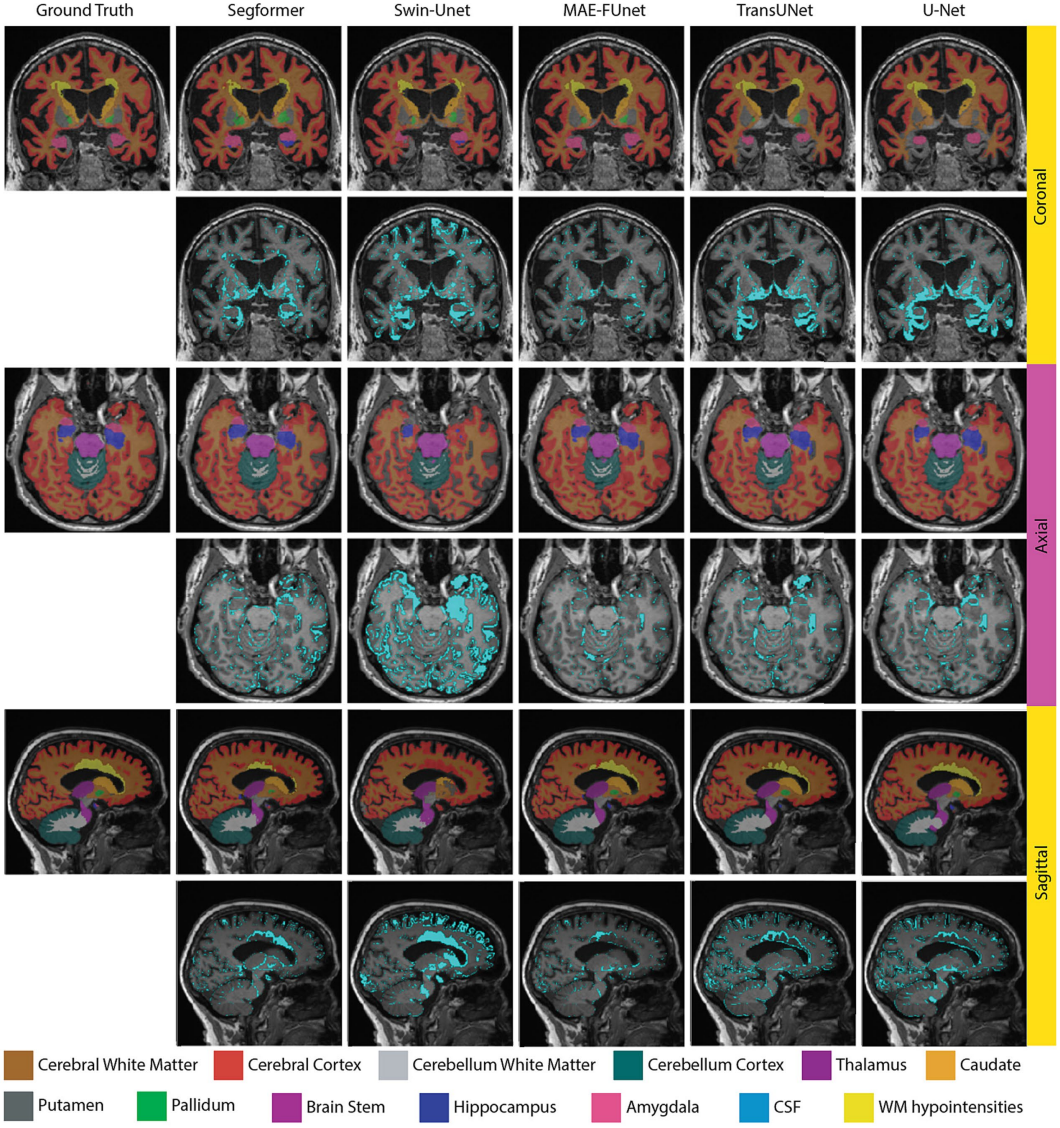
Qualitative segmentation results trained on 30 samples from the NACC dataset, demonstrated by sagittal, coronal, and axial views on each row. For each row, the top includes predicted brain region masks, and the bottom includes error maps with cyan highlighting mislabeled pixels.

Qualitative comparisons across different models are provided in Figure 10, including sampled sagittal, coronal, and axial views of 13 anatomical regions in the NACC dataset. Each view displays segmentation predictions in the top row and corresponding error maps in the bottom row. In general, MAE-FUnet demonstrates improved anatomical accuracy with fewer boundary artifacts and more consistent segmentation of complex regions. Consistent with the results observed in the skull stripping task, MAE-FUnet produces sharper, cleaner boundaries than other models, particularly in regions with narrow and elongated anatomical contours such as the lateral ventricles. Furthermore, MAE-FUnet is superior in capturing small regions such as the hippocampus, amygdala, and putamen, while other models either under-segment (e.g., missing the amygdala) or over-segment (e.g., spilling over into neighboring tissue). The error maps further indicate MAE-FUnet’s improved performance, with visibly fewer mislabeled pixels, as reflected by reduced cyan overlays compared to other baselines. In summary, both qualitative and quantitative indicate higher segmentation fidelity for MAE-FUnet, particularly for complex or small anatomical regions. By fusing hierarchical CNN features with learned MAE embeddings, the proposed model preserves both local detail and global consistency in few-shot learning, whereas other baselines often struggle.

### 3.4 Ablation study

To further investigate the key design components in MAE-FUnet, we perform ablation experiments focusing on two major aspects: (1) the impact of model sizes controlled by initial channel dimension, and (2) the effect of different fusion strategies, including addition, concatenation, and attention.

In Table 9, we evaluate three MAE-FUnet variants: MAE-FUnet32, MAE-FUnet64, and MAE-FUnet96, corresponding to initial convolutional channel dimensions of 32, 64, and 96. All other architectural and training configurations remain identical to those described in Sections 3.2 and 3.3. For this ablation study, the training data are fixed at a slice sampling stride of 7 for NFBS and SynthStrip, 7 training volumes for MRBrainS18, and 30 training volumes for NACC. By comparing all benchmarks, MAE-FUnet64 achieves the best performance on NFBS and SynthStrip for skull stripping, while MAE-FUnet96 attains the highest Dice and IoU scores on MRBrainS18 and NACC for multi-class segmentation.

**Table 9:** Ablation study on different model sizes controlled by initial feature dimension in MAE-FUnet across skull stripping and multi-class segmentation tasks.

Method	IoU/Dice (%)				Trainable params
	Skull strip		Multi-class segmentation		
	NFBS	SynthStrip	MRBrainS18	NACC	
MAE-FUnet32	96.15/98.04	95.21/97.55	71.27/82.19	70.62/82.08	10 M
MAE-FUnet64	**96.57/98.26**	**95.25/97.57**	72.41/83.06	72.88/83.70	38 M
MAE-FUnet96	96.27/98.10	95.19/97.53	**73.21/83.64**	**73.27/83.85**	84 M

The performance is recorded by overall IoU and Dice loss on each dataset, along with the number of trainable parameters in millions (M). Bold values indicate the best performance among all compared methods for each metric.

Increasing the channel dimension from 32 to 64 improves performance on both skull stripping and multi-class segmentation. This trend suggests that richer low-level feature representations are crucial for capturing anatomical context. However, further increasing the dimension to 96 does not yield consistent improvements and can result in marginal performance drops on simpler tasks such as skull stripping. For instance, on the NFBS dataset, the IoU decreases by 0.3% from MAE-FUnet64 to MAE-FUnet96, despite more than doubling the parameter count from 38 M to 84 M. In contrast, for more complex anatomical segmentation tasks presented in MRBrainS18 and NACC, performance generally benefits from increased model capacity. These findings highlight an important consideration for few-shot learning: *increasing model complexity does not universally translate to better performance.* Instead, the optimal model configuration can be influenced by the complexity of the target task, and balanced architectural design is essential under data-limited conditions.

In Table 10, we conduct an ablation study comparing different fusion mechanisms for integrating MAE transformer embeddings with CNN features. Specifically, we compare three strategies: additive fusion (MAE-FUnet-add), concatenation fusion (MAE-FUnet-concat), and attention-based fusion (MAE-FUnet-attent). All other architectural and training configurations are kept identical to those described in Sections 3.2 and 3.3, with the initial convolutional channel dimension fixed at 64. Among these three strategies, the concatenation approach achieves the best performance by attaining the highest Dice and IoU scores across all four datasets in both skull stripping and anatomical segmentation tasks, while maintaining a moderate parameter size (38 M). MAE-FUnet-attent provides slight improvements in some cases over the additive strategy but consistently underperforms MAE-FUnet-concat. In fact, attention-based strategy adds more computational overhead without consistent performance gains. Overall, these results indicate that concatenation is the most effective fusion strategy, as it preserves and combines complementary information from CNN features and transformer embeddings, thereby improving spatial integration and segmentation accuracy.

**Table 10:** Ablation study on fusion strategies—addition, concatenation, and attention—in MAE-FUnet across skull stripping and multi-class segmentation tasks.

Method	IoU/Dice (%)				Trainable params
	Skull strip		Multi-class segmentation		
	NFBS	SynthStrip	MRBrainS18	NACC	
MAE-FUnet-add	95.73/97.82	95.20/97.54	71.62/82.52	72.35/83.36	35 M
MAE-FUnet-concat	**96.57/98.26**	**95.25/97.57**	**72.41/83.06**	**72.88/83.70**	38 M
MAE-FUnet-attent	95.99/97.96	95.23/97.56	71.60/82.51	71.59/82.83	41 M

The performance is reported as overall IoU and Dice loss for each dataset, along with the number of trainable parameters in millions (M). Bold values indicate the best performance among all compared methods for each metric.

## 4 Discussion

This study presents a systematic investigation into the few-shot deployment of pretrained MRI transformers for diverse, non-pathological brain imaging tasks, including both high-level tasks such as classification and low-level tasks such as segmentation. Using a Masked Autoencoder pretraining framework, we demonstrate that highly transferable latent representations can be learned from a large-scale, multi-cohort, unlabeled brain MRI corpus comprising over 31 million 2D slices. These representations can be effectively reused across multiple downstream tasks, even under data-scarce conditions. Our study shows that pretrained MAE transformers offer strong generalizability across tasks, modalities, and datasets, without requiring extensive data preprocessing steps such as MRI registration, bias field correction, or reorientation (Palumbo et al., 2019), nor complex fine-tuning procedures. Thus, it is particularly suitable for fast deployment in resource-constrained research and clinical pipelines.

For classification tasks such as MRI sequence detection, we find that a simple linear classifier appended to a frozen MAE encoder is sufficient to outperform well-established architectures, including ResNet and EfficientNetv2. This highlights the strength of the learned MAE representations and their ability to capture semantic content with minimal task-specific adaptation. When evaluated under controlled few-shot training conditions, the classification model yields strong performance across multiple limited-data regimes, demonstrating the data efficiency and scalability of the proposed framework. Additionally, the small number of trainable parameters in the classification head highlights the lightweight design and potential for rapid deployment in practice.

For segmentation, we introduce MAE-FUnet, a hybrid architecture that fuses multi-scale CNN features with transformer embeddings from a pretrained MAE encoder, designed for flexible deployment and adaptation across tasks. Evaluations on skull stripping and multi-class anatomical segmentation over the NFBS, SynthStrip, MRBrainS18, and NACC datasets demonstrate that MAE-FUnet achieves competitive accuracy, improved few-shot robustness, and lower performance variance compared with other segmentation baselines such as TransUnet and SegFormer. Ablation studies further demonstrate that optimal performance relies on model capacity, fusion strategy, and appropriate alignment with task complexity. These findings suggest that suitable architectural design is essential when integrating pretrained transformers with other machine learning modules, especially under data-scarce conditions.

Notably, to emphasize the generalization ability of the pretrained MAE encoder, we focus on 2D MRI slices instead of 3D MRIs. This 2D-based design enables inclusion of a broader range of MRI data, including fast MRI acquisitions, scans with large slice thickness, and single-slice images available in public datasets such as RadImageNet. Because transformer pretraining is data-intensive, we prioritize expanding the pretraining dataset scale over learning 3D correlations in MRI scans. However, as public 3D MRI cohorts continue to accumulate, adapting our pretraining on 3D MRI will become a promising target. Another potential bias in our framework is the demographic distribution within the three major brain MRI cohorts. NACC, ADNI, and OASIS are primarily collected from older patient groups with potential dementia-related pathology. Although our experiments focus only on non-pathological tasks, the overrepresentation of aging populations may still limit the generalization of the pretrained MAE encoder.

Nevertheless, our findings emphasize the importance of designing frameworks that fully leverage the potential of learned MAE embeddings while carefully balancing simplicity of adaptation and model complexity. In particular, we demonstrate that integrating latent-space embeddings with other architectural modules, such as CNNs, is effective for medical image segmentation. Although CNNs excel at capturing local image patterns, they often struggle with global spatial coherence. Meanwhile, MAE-derived embeddings provide a rich, hierarchical understanding of the image at various scales. By combining both modules, our MAE-FUnet framework aims to effectively integrate local details with global context. This hybrid approach is well-suited for medical imaging tasks, where accurate delineation of fine-grained anatomical structures and high-level spatial reasoning are both critical. Furthermore, the adaptability and scalability of the MAE-FUnet provide greater flexibility for various downstream tasks with diverse objectives. While this study focuses on classification and segmentation, the same fusion paradigm could potentially be extended to image-to-image enhancement tasks, such as denoising, super-resolution, or artifact correction.

In conclusion, our work not only provides a practical, parameter-efficient pathway for few-shot medical AI with pretrained transformers, but also offers a generalizable, reproducible fusion-based architecture design pipeline. Moving forward, we expect that our framework can be expanded to multi-modal brain imaging (e.g., MRI + PET), cross-domain generalization (utilization in other body locations), and downstream clinical applications such as pathology detection and disease progression prediction (Grueso and Viejo-Sobera, 2021; Kamal et al., 2025). As transformer architectures continue to evolve and large-scale medical imaging datasets accumulate, this work lays the foundation for scalable, data-efficient, clinically deployable AI systems for medical imaging.

## Data Availability

The ADNI dataset used in the current study is available on the Alzheimer's Disease Neuroimaging Initiative office website at: https://adni.loni.usc.edu/. To access the ADNI data repository, one must register with a valid account and complete the online application process. The NACC dataset used in the current study is available on the National Alzheimer’s Coordinating Center office website at: https://naccdata.org/requesting-data/data-request-process. To access the NACC data repository, registration with a valid account is required, followed by the online application process. The OASIS dataset used in the current study can be applied in the Open Access Series of Imaging Studies office website at: https://sites.wustl.edu/oasisbrains/. To access the OASIS data repository, registration with a valid account is required, followed by the online application process. The RadImageNet dataset used in the current study can be accessed by creating an account on the RAD IMAGE NET official website at: https://www.radimagenet.com/. To access the RadImageNet data repository, registration with a valid account is required, followed by the online application process. The fastMRI dataset used in the current study can be applied on the NYU fastMRI official website. To access the fastMRI data repository: https://fastmri.med.nyu.edu/, you need to submit an online application and a valid email address to receive downloadable links. The NFBS dataset used in the current study can be directly accessed in the NFBS Skull-Stripped Repository: http://preprocessed-connectomes-project.org/NFB_skullstripped. The NFBS repository is publicly available and can be downloaded from the webpage. The SynthStrip dataset used in the current study can be directly accessed in the SynthStrip: Skull-Stripping for Any Brain Image Repository: https://surfer.nmr.mgh.harvard.edu/docs/synthstrip/. SynthStrip repository is publicly available and can be downloaded on webpage. The MRBrainS18 dataset used in the current study can be directly accessed in the MR Brain Segmentation at MICCAI 2018 Repository: https://mrbrains18.isi.uu.nl/data/download/index.html. MRBrainS18 repository is publicly available and can be downloaded on webpage. All data/code and the pretrained models generated in this study are accessible through Github Repository: https://github.com/MengyuLiGit/MAE-FUnet-MRI-finetune.
