# Supplementary material for: Few-shot deployment of pretrained MRI transformers in brain imaging tasks
Source: Front Artif Intell. 2026 Apr 1;9:1771088. doi: 10.3389/frai.2026.1771088 (PMC13079346; doi:10.3389/frai.2026.1771088)
Supplement: Supplementary file 1 [file Data_Sheet_1.PDF]

## Supplementary Information

### S.1 Pretrain Mathematics

**Loss function:**

The loss function used in this study introduces a sample-wise weighting scheme to address partial brain coverage. As the original MAE, the function first computes per-sample loss, averaged over masked patches:

$$\tilde{l}_i = \frac{\sum_{j=1}^{L} m_{ij} l_{ij}}{\max\left(1, \sum_{j=1}^{L} m_{ij}\right)} \quad (S1)$$

Where $m_{ij} \in \{0, 1\}$ is the binary indicator for whether the patch $j$ in sample $i$ is masked. $l_{ij}$ is the mean squared error (MSE) for the patch $j$ in sample $i$.

For the whole batch with $N$ samples, we compute the final loss as the weighted average of the per-sample losses:

$$\mathcal{L} = \frac{1}{N} \sum_{i=1}^{N} w_i \tilde{l}_i \quad (S2)$$

where $w_i$ is a sample-specific weighting factor based on the brain area coverage of the sample $i$. Let $a_i$ denote the number of masked pixels within the brain region of the sample $i$, and define the set of low-coverage samples as $S = \{j | m_j < \tau\}$, where $\tau$ is a pre-defined threshold for sufficient brain area.

The weighting function is defined as:

$$w_i = \begin{cases} 1, & if\ a_i \geq \tau \\ \left( \frac{a_i + \beta}{\max_{j \in S}\left(a_j + \beta\right)} \right)^{1.2} & if\ a_i < \tau \end{cases} \quad (S3)$$

where β is a baseline weight (e.g., 8000) that prevents the weight from collapsing for MRI with no brain area. This formulation ensures that samples with limited brain content are down-weighted proportionally while still contributing to the loss in a stable manner. In our setting, this results in a minimum normalized weight clamped above 0.2.

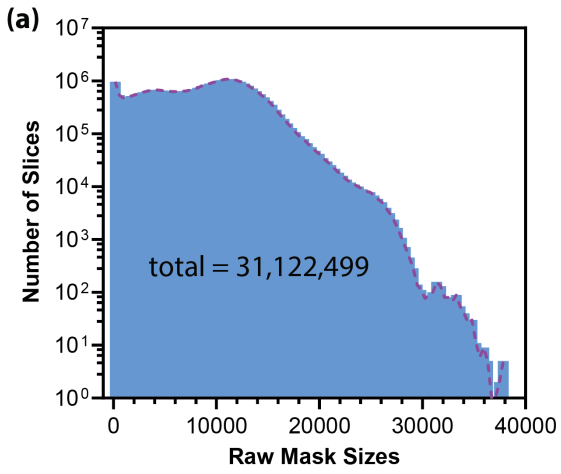


**Figure S1.** (a) Histogram of raw brain mask sizes (number of pixels per mask) across all training slices, reflecting the diversity in brain coverage. (b) Normalized sample weights derived from brain region coverage, used to down-weight low-coverage slices during training.

To address the variation in anatomical content present across aggregated MRI datasets, we provide an analysis of the distribution of brain region coverage and its impact on loss weighting. Figure S1(a) demonstrates the distribution of raw brain mask sizes in all 2D slices, which presents the variation in the brain mask content, from intact anatomical views to severely cropped or empty regions. By scaling each sample's loss with respect to the brain coverage of the corresponding slice, we aim to reduce the impact of low-coverage slices during training. We depict these normalized weights in Fig. S1(b), where samples that are fully covered still receive full weight (1.0), while lower-covered samples are smoothly down-weighted. Given the configurations set in Equation S3, this mechanism intends to enable stable optimization and prevents the model from overfitting to non-informative or truncated input. The training is conducted 30 epochs on the overall dataset with 256 batch sizes using the AdamW optimizer [57] and $1 \times 10^{-4}$ learning rate.
